# The impact of tethered recording techniques on activity and sleep patterns in rats

**DOI:** 10.1038/s41598-022-06307-3

**Published:** 2022-02-24

**Authors:** Katharina Aulehner, Jack Bray, Ines Koska, Claudia Pace, Rupert Palme, Matthias Kreuzer, Bettina Platt, Thomas Fenzl, Heidrun Potschka

**Affiliations:** 1 Institute of Pharmacology, Toxicology, and Pharmacy, Ludwig-Maximilians-University, Königinstr. 16, 80539 Munich, Germany; 2 School of Medical Sciences, Institute of Medical Sciences, University of Aberdeen, Foresterhill, Aberdeen, Scotland UK; 3 Department of Biomedical Sciences, University of Veterinary Medicine, Vienna, Austria; 4 Department of Anesthesiology and Intensive Care, School of Medicine, Technical University of Munich, 81675 Munich, Germany

**Keywords:** Biological techniques, Neuroscience

## Abstract

Electrophysiological recordings in animals constitute frequently applied techniques to study neuronal function. In this context, several authors described tethered recordings as a semi-restraint situation with negative implications for animal welfare and suggested radiotelemetric setups as a refinement measure. Thus, we here investigated the hypothesis that tethered recordings exert measurable effects on behavioral and sleep patterns in Sprague–Dawley rats. Animals were kept in monitoring glass cages either with or without a head connection to a recording cable. Saccharin preference, nest building, serum corticosterone and fecal corticosterone metabolite levels were in a comparable range in both groups. The proportion of vigilance states was not affected by the cable connection. Minor group differences were detected in bout lengths distributions, with a trend for longer NREM and WAKE stages in animals with a cable connection. However, a relevant effect was not further confirmed by an analysis of the number of sleep/wake and wake/sleep transitions. The analysis of activity levels did not reveal group differences. However, prolonged exposure to the tethered condition resulted in an intra-group increase of activity. In conclusion, the comparison between freely moving vs tethered rats did not reveal major group differences. Our findings indicate that telemetric recordings only offer small advantages vs cabled set ups, though this may differ in other experimental studies where for example anxiety- or drug-induced effects are analyzed.

## Introduction

In 1959 Russel and Burch have introduced the 3R principle, which provided a framework for more humane experimental techniques^1^. Refinement is based on experimental measures and techniques aiming to minimize pain, distress, suffering or lasting harm of animals. While the relevance for animal welfare is a matter of course, there is also an increasing awareness that minimizing the burden for the animals is of relevance for data quality^2,3^.

Decisions about potential refinement measures are often taken from an anthropocentric point of view without scientific evidence for the efficacy of the respective measure. Thus, it is of utmost relevance to empirically improve our knowledge about the impact of procedures and techniques on the welfare of laboratory animals.

In neuroscience research, electrophysiological recordings constitute frequently applied techniques as they enable functional analyses of brain signaling. Transmission of electrographic data can be either based on cables or on radiotelemetric setups^2–4^. A connection of a headmount with a technical set up is also required for electrical or optical stimulation, as well as prolonged intracerebral drug delivery or extracellular fluid sampling techniques. Thereby, torque on the headmount and the associated movement constraint needs to be taken into account concerning animal welfare and data quality considerations^2^.

The optimization of tethered systems with swivels, adjusted length of the cable and the integration of counterweights or springs can limit the impact on the animal’s free movement^2^, but the question remains to what extent the remaining influence of restraint may compromise the animal’s wellbeing. Several authors have described tethered recordings as a semi-restraint situation with negative implications for animal welfare^2–6^. This question is of particular relevance when it comes to the weighing of different technical options. In the context of long-term recordings it has been suggested that radiotelemetry, despite the need for transmitter implants, may have benefits for animal welfare^2–4,6^.

In addition to animal welfare considerations, the effects of applied techniques on readout measures need to be taken into account for study design and data interpretation. Awareness of confounding factors is of particular relevance in the context of rigor and reproducibility in animal-based research^7,8^.

Recently, we reported that behavioral alterations characterizing post-status epilepticus models differ between animals with telemetric and tethered recordings^9,10^. These findings are of particular interest for the interpretation of behavioral data and the design of studies in chronic disease models. However, conclusions concerning associated distress were not clear cut, considering the behavioral patterns observed in our studies.

We therefore set out to further analyze the impact of tethered recordings on animals’ wellbeing based on a detailed analysis of activity and sleep patterns. The experimental approach sought to address our hypothesis that tethered recordings exert relevant effects on activity, rest and sleep patterns in rats.

## Material and methods

### Animals

A total of fourteen female Sprague Dawley rats (190–210 g; 9–11 weeks; Envigo, Horst, The Netherlands) were used for this study. Animals were single housed under controlled environmental conditions (22–24 °C, 45–65% relative humidity) in a 12 h:12 h light/dark cycle (lights on between 5 a.m. and 5 p.m.) with tap water and pelleted food (Ssniff Spezialdiäten GmbH, Soest, Germany) available ad libitum. Rats received a clean Makrolon type III cage with bedding material (Lignocel, Rosenberg, Germany) and 14 g of nest material (Enviro Dri, Claus GmbH, Neuwied Germany) weekly. Animal IDs were randomly assigned on arrival by simple randomization using R, version 3.6.1 via RStudio, version 1.2.1335. The study was approved by the government of Upper Bavaria (reference number ROB-55.2-2532.Vet_02-16-105) and was conducted in line with the German Animal Welfare act and the EU directive 2010/63/EU. All the procedures were performed and all the data are reported according to the ARRIVE guidelines and the Basel declaration including the 3R concept. The severity of the procedure was classified as moderate pro- and retrospectively.

### Study design

Rats were randomly allocated to two experimental groups (nontethered, n = 7; tethered, n = 7) via stratified randomization using R, version 3.6.1. via RStudio, version 1.2.1335. The stratified randomization was conducted based on body weight 1 week prior to the telemetric recordings.

After arrival, animals were allowed to habituate for one and a half weeks prior to surgery. During habituation, each rat was gently handled twice per day to reduce stress and to habituate to the investigator. Additionally, baseline data for rat grimace scale (RGS), Irwin score and nest building were assessed during the habituation phase and feces were collected (see details below). After 3.5 weeks of recovery, the animals were placed in individual modified plexiglass cages (800 cm^2^) for 14 days where the electroencephalographic (EEG), electromyographic (EMG) and activity recordings were conducted (see details below). The rats in the tethered group were connected to a cable (weight: 8.5 g, length: 42 cm) equipped with a non-motorized swivel system (Supplementary Fig. S1). The cable was plugged into a fixed point on the lid of the glass chamber. The swivel-tether system enabled the cable to rotate, allowing free movement within the glass chamber. As recordings from both groups were obtained from the implanted telemetric transmitters, the tethering cable served no recording function. One day after the experimental observation period, the animals were euthanized with intraperitoneal sodium pentobarbital injection (600 mg/kg Narcoren^®^, Merial GmbH, Hallbergmoos, Germany). Metamizole (100 mg/kg, Vetalgin^®^, Covetrus, Germany) was administered perorally to prevent pain during euthanasia. Blood samples for serum corticosterone measurements were collected by cardiac puncture. Please note that the study was designed to also obtain important information about the habituation to the cable, so that recordings were immediately started following the first connection to the cable to assess the course of any alterations during the entire exposure phase.

**Figure 1:**
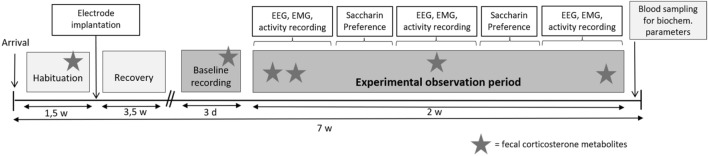
Timeline of the study. EEG: electroencephalographic; EMG: electromyographic.

The researcher was not blinded during the experiment due to the clearly visible cable; however, the researchers were blinded during the sleep pattern evaluation and the evaluation of the biochemical data. The timeline of the study is described in Fig. 1.

### Surgical procedure

The animals were implanted with EEG and EMG electrodes. For detailed description of the surgical procedure see Supplementary Methods S1.

### Clinical score, Irwin score, Grimace score, body weight analysis

The rat grimace scale (RGS)^11^ and the Irwin score^12^ were evaluated prior to surgery and on Day 1, Day 2, Day 3 and Day 4 post-surgery until values returned to baseline. The RGS action units were averaged for each animal per day and the mean difference scores were analyzed by subtracting the baseline. For the evaluation of the Irwin score, the total score for each animal per day was calculated as the sum of all evaluated parameters, not considering the signs (minus and plus), i.e. considering all deviations from normal. In accordance with the allowance, animals were intensively monitored using the standard clinical score. Body weights were monitored at least weekly throughout the study.

### Telemetric recordings

Three channels, one for activity, one for EMG, and one for EEG, were used in order to record the data of interest. During baseline recording, the home cage of each rat was placed on a DSI telemetry receiver (DSI, St. Paul, USA) that was connected to an acquisition computer. During the experimental observation period, the telemetry receiver was placed under the glass chambers. The telemetric transponders (HD-X02, 2.2 g, 1.7 cc) were activated for baseline telemetric recordings on three consecutive days after the recovery phase and for nontethered/tethered recordings on 9 days during the 14-day experimental observation phase. Telemetric data were continuously recorded each day for 23 h, with one hour per 24 h considered for animal maintenance. The EEG and EMG data were sampled at 1000 Hz and the activity data were sampled at 50 Hz. The telemetric recordings were performed using Ponemah software (Ponemah R, v. 5.2.0, DSI, St. Paul, USA).

One animal from the tethered group was euthanized during the recovery phase due to wound healing issues and was not considered for telemetric recordings.

### Analysis of sleep and activity patterns

Sleep and activity patterns were analyzed according to Crouch et al.^13^. We here applied standard Fast Fourier Transformation (FFT) spectral decomposition, in line with the most commonly applied methods in the field. Briefly, for each recording day, the telemetric data were converted into EDF format for further analysis with MATLAB R2020a (MathWorks Inc, USA). The raw data were converted into hourly epochs and band pass filtered between 0.75 and 45 Hz. The data were then segmented into 10 s epochs. Each epoch was scored by using FFT spectral bands analysis and assigned to one of four vigilance stages: quiet awake (QWAKE), active awake (AWAKE), NREM sleep, and REM sleep. The respective scoring values were: 0 (AWAKE), 1 (QWAKE), 2 (NREM sleep), or 3 (REM sleep). Artefact removal rejected any epochs that had power greater than the specified threshold (400 mV). EEG frequency bands of interest were defined according to Mondino et al.^14^ as Delta (1–5 Hz) and Theta (5–9 Hz). If there was a high variance in the EMG within an epoch, the epoch was scored as WAKE. The activity data as recorded via Ponemah, were used to score epochs as AWAKE. All other epochs scored as WAKE were assigned to QWAKE. An epoch was scored as REM sleep if the theta to delta band power ratio was more than 1.75 whilst the animal was inactive. If there was a high delta frequency and EMG and activity were low, the epoch was scored as NREM sleep. Because artifacts in the recorded signals led to a number of invalid scores, the recordings were rescored manually. Because of technical issues in the last 2 h of the recording in some animals and maintenance time, we only performed the analyses on the first 22 h (Zeitgeber time 0–22) of each day.

### Analysis of sleep architecture

For assessment of the general sleep architecture we derived the following information from the scoring vectors: (i) the proportion of the vigilance states for nonoverlapping 2 h observation episodes throughout the 22 h, (ii) the bout length distribution of each vigilance state, and (iii) the number of transitions between wake and sleep, NREM sleep and REM sleep, and QWAKE and AWAKE in the light or dark period. We focused on the proportions of vigilance states to check for differences between the two groups at baseline conditions. When investigating possible differences during the 2-week experimental observation period we focused on the 1st day, the first 3 days (early), and the last 3 days (late).

### Quantitative EEG analysis slow-wave activity power

The slow-wave activity (SWA) power during NREM sleep indicates sleep quality, depending on prior awaking^15^. This means that the duration of the preceding wakefulness is one of the main factors influencing subsequent total sleep duration and, in particular, sleep quality^15,16^. Therefore, we calculated the SWA power for each animal and each day by calculating the power spectral density (PSD) for each 10-s episode that was scored NREM. We defined the SWA power as the cumulative power from 0.5 to 4 Hz. These values were averaged for each animal for the dark and light period.

### Analysis of home cage behavior and saccharin preference

Nest building assessment was performed according to previous studies^17,18^ in our facility. Briefly, nest building photos were taken daily 1 to 3 h after the onset of the light phase in the week before the surgery and during the experimental observation period. Based on the photos, the complexity of the nest was rated with a score between 0 and 4.

The saccharin preference test was performed according to previous studies^18,19^, with one test shortened from 4 to 2 days in order to avoid distracting the animals with saccharin during telemetric recordings. Briefly, on the first day, two water bottles were provided per cage and the water intake from both bottles over 24 h was evaluated. On the second day, the bottle on the right side was filled with 0.1% saccharin solution (Aldrich Saccharin ≥ 98%, Sigma-Aldrich Chemie GmbH, Germany). The intake of water and saccharin solution was determined for 24 h.

### Analysis of fecal corticosterone metabolites (FCMs) and serum corticosterone

Baseline fecal samples were collected during the habituation phase and the baseline telemetric recordings. Since distress is reflected in corticosterone metabolite levels after 12 to 14 h, as described by Lepschy et al.^20^, we collected fecal samples on day 1 and day 2 of the experimental observation period. To evaluate the influence of a potential habituation, we additionally collected fecal samples on day 8 and 14 of the experimental observation period. Feces were collected at the same time at 7 p.m. At the end of the study, serum was collected for the determination of serum corticosterone levels. Analyses of fecal corticosterone metabolites and serum corticosterone were performed as described previously by Möller et al.^18^ and Lepschy et al.^20^.

### Analysis of activity count

To assess the impact of the cable on activity (distance moved, speed) the activity signal recorded with Ponemah was evaluated. The activity, i.e. the movement of the animal in the cage, was generated in counts per minute (cpm) by the change of the distance of the telemetric transponder to the telemetry receiver containing the antennas. When the animal moves across the telemetry receiver, a count number was generated depending on the distance covered and speed. For the assessment of activity level, the total activity per animal was averaged for nonoverlapping, consecutive 2 h observation episodes throughout the 22 h.

### Statistical analysis

An a priori power analysis was conducted based on existing literature data using the statistical power analysis program G*Power 3.1.9.4.0 to determine the sample size for this study. For this, the percentage of REM sleep per day was considered as the main outcome measure. The relevant difference was set at 2% REM sleep difference. Data (means ± SEM) for REM sleep, from a sleep deprivation group and from a control group, were taken from Table 1 of Guzman-Marin et al.^21^. Calculation of the effect size resulted in a value of 2. A sample size of 6 animals per group was required to determine a significant difference between the two groups with a significance level of 0.05 and a power of 0.80. Based on nonnormal distribution found with the Shapiro–Wilk-Test and the rather small sample size per group, we decided to perform nonparametric tests, except for FCMs, serum corticosterone, activity counts, and body weight.

For the evaluation of possible differences between the tethered and nontethered rats in the proportion of the vigilance states over the 22 h observation period, we calculated the area under the receiver operating characteristic (AUC) together with 10,000-fold bootstrapped 95% confidence intervals. A 95% confidence interval (CI) exclusive 0.5 indicates a significant difference^22^. For AUC calculation we used the measures-of-effect-size-toolbox for MATLAB^22^. In order to avoid false positives due to multiple comparisons, we only describe significant differences if they occur in two neighboring 2 h episodes. This approach has been used in similar fashion before^23,24^. For the evaluation of differences in transition frequencies and in SWA power between the groups we used the Mann–Whitney-U test (two-tailed). We tested for differences in the distribution in bout lengths between the tethered and nontethered rats using the Kolmogorov–Smirnov test and cumulative probability plots. This approach has also been previously employed in sleep research^25,26^. In general, we checked for differences in the parameters between the groups using averaged values for the 3-day baseline, for the first (early) or last (late) 3 days of the experimental observation period as well as for the first day of the experimental observation period.

We analyzed nest building scores and anhedonia-associated behavior using the Mann–Whitney-U test (two-tailed). A two-way ANOVA with factors “nontethered/tethered” and “days”, followed by a post-hoc Bonferroni multiple comparison test was conducted for the assessment of FCMs between the two groups within the experimental observation period. An unpaired t-test (two-tailed) was applied to analyze FCMs averaged over all experimental observation days and to analyze corticosterone serum levels. To assess the body weight a two-way ANOVA with factors “nontethered/tethered” and “time”, followed by a post-hoc Bonferroni multiple comparison test was conducted. A two-way ANOVA with factors “nontethered/tethered” and “hours”, followed by a post-hoc Bonferroni multiple comparison test was conducted for the assessment of activity counts between the two groups during the experimental observation period. Activity, behavioral and biochemical parameters were analyzed using GraphPad Prism (v5.04). A P value of less than 0.05 was considered statistically significant.

AUC values and 10,000-fold bootstrapped 95% confidence intervals were calculated for sleep/wake transitions, SWA power, behavioral and biochemical parameters’ to complete the statistical comparison. A linear regression model for the proportions of vigilance states, activity, and other behavioral parameters was constructed with the MATLAB fitlm function to test for possible within-group habituation effects throughout the observation period.

## Results

### Impact of surgical procedures

In the early phase following surgery, a slight increase in the RGS was evident. The score reached a peak at the first post-surgical day and then steadily decreased until the fourth day following surgery. The maximum did not exceed a mean difference score of 0.421 (score from minimum 0 to maximum 2; Supplementary Fig. S2).

The early post-surgical phase was also characterized by a slight increase of the total Irwin score reaching a maximum of 1.07 (score from minimum 0 to maximum 61) at the first post-surgical day, and a return to normal levels at day three following surgery (Supplementary Fig. S2). The parameters affected as a consequence of the surgical procedure included piloerection and locomotor activity. Respective alterations were also captured by the standard clinical score evaluated on a daily basis in accordance with the allowance approved by the local government (data not shown). No group differences became evident for body weight development during the observation period (Supplementary Fig. S2).

### Impact of the tethering cable on the proportion of vigilance states and on sleep microarchitecture

**Figure 2:**
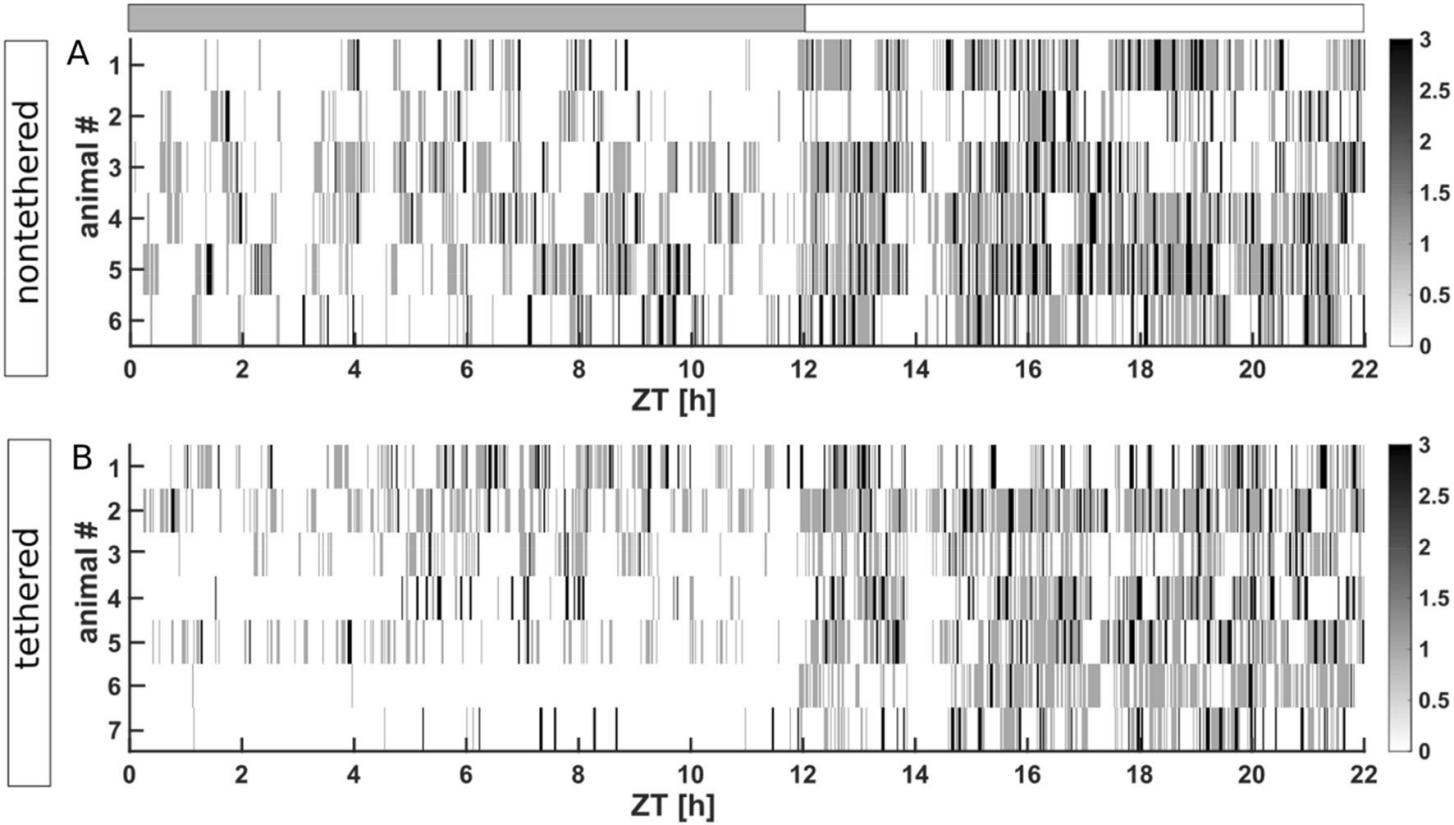
Hypnogram of the nontethered (**A**) and tethered (**B**) animals throughout the 22 h experimental observation period. Lights were turned on after 12 h. The x-axis represents Zeitgeber time (ZT). The gray rectangle exhibits the dark phase, the white rectangle exhibits the light phase. White exhibits WAKE, light gray exhibits NREM sleep and dark gray exhibits REM sleep. The synchronous interruption of sleep at H14 (ZT) coincides with entering the animal room to take nest photos. This synchronous interruption at this time of the day is visible throughout the entire experimental observation period. #: individual animal number.

Hypnograms from both experimental groups are illustrated in Fig. 2 (observation period) and Supplementary Fig. S3 (3rd day of baseline recording). In both groups the characteristic nocturnal patterns are evident with longer periods of WAKE and brief periods of sleep during the dark phase. Frequent disruptions of sleep periods were evident during this time of the day. During the light phase, animals exhibited repeated cycling between NREM and REM sleep stages and brief WAKE periods. Only a limited number of longer WAKE periods occurred during this phase. One of the tethered animals exhibited a reduction of sleep episodes during the dark and light phase (Fig. 2B; animal #6).

The proportion of vigilance states for nonoverlapping 2 h observation episodes throughout the 22 h observation period was analyzed considering all baseline days. During these baseline recordings the proportion of vigilance states (WAKE, NREM sleep, REM sleep) was not significantly different between animals later on allocated to the groups with and without a cable connection (Supplementary Fig. S4).

The proportion of vigilance states for nonoverlapping 2 h observation episodes throughout the 22 h observation period was analyzed considering all experimental observation days. In addition, we separately analyzed data from day 1, the first three, and the final 3 days of the observation period, at the beginning, and at the end of the phase in the glass monitoring cages.

**Figure 3:**
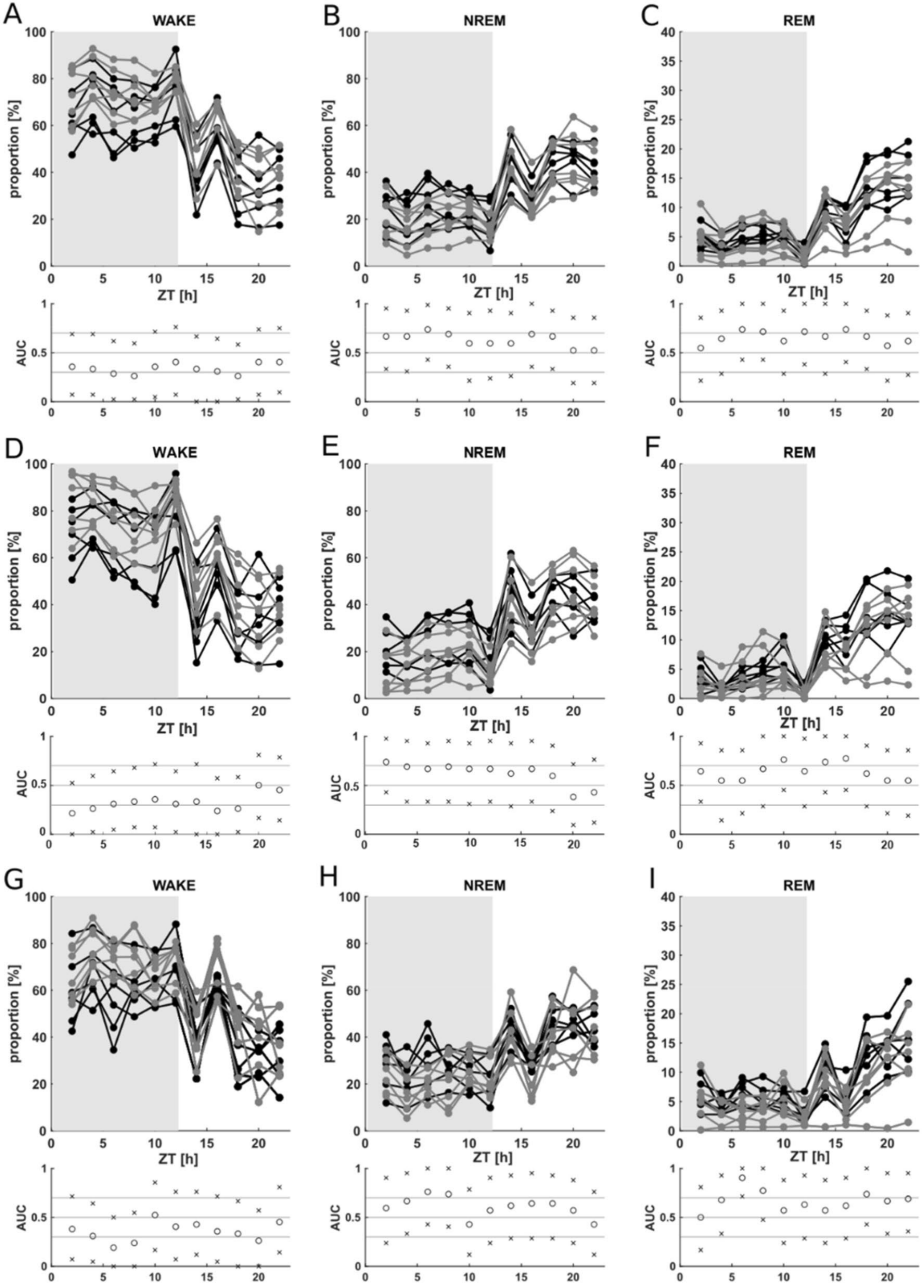
Proportion of the vigilance states of the nontethered (black, n = 6) and tethered (gray, n = 7) animals for nonoverlapping 2 h observation episodes throughout the 22 h. Lights were turned on after 12 h. Gray exhibits the dark phase, white exhibits the light phase. The x-axis represents Zeitgeber time (ZT). Circles represent the calculated AUC. The limits of the 95% confidence intervals are represented by the letter x. NREM: NREM sleep, REM: REM sleep (**A**–**C**) Averaged values for all 9 days of the experimental observation period for WAKE (**A**), NREM sleep (**B**) and REM sleep (**C**). The AUC analysis detected no effect between the nontethered (black) and tethered (gray) animals. (**D**–**F**) Averaged values for the first 3 days (early) of the experimental observation period for WAKE (**D**), NREM sleep (**E**) and REM sleep (**F**). The AUC analysis detected no effect between the nontethered (black) and tethered (gray) animals. (**G**–**I**) Averaged values for the last 3 days (late) of the experimental observation period for WAKE (**G**), NREM sleep (**H**) and REM (**I**) sleep. The AUC analysis detected no effect between the nontethered (black) and tethered (gray) animals. Data are plotted individually for each animal.

The proportion of vigilance states (WAKE, NREM sleep, REM sleep) was not significantly different between animals with and without a cable connection at any time point (Fig. 3, Supplementary Fig. S4).

We additionally calculated the linear regression for the proportion of vigilance stages for each animal averaged per day throughout the observation phase (Supplementary Fig. S5). The proportion of active WAKE was not significantly different over the time course of the observation period, both within the tethered group and within the nontethered group. In both groups, we detected a significant decrease in quiet WAKE (nontethered: p = 0.014, tethered: p = 0.028) and a significant increase in NREM sleep (nontethered: p = 0.045, tethered: p = 0.020) from the early to the late experimental observation phase. The proportion of REM did not differ significantly over the time course of the observation period, both within the tethered group and within the nontethered group.

### Impact of the tethering cable on bout length and on transition between wake and sleep phases

**Figure 4:**
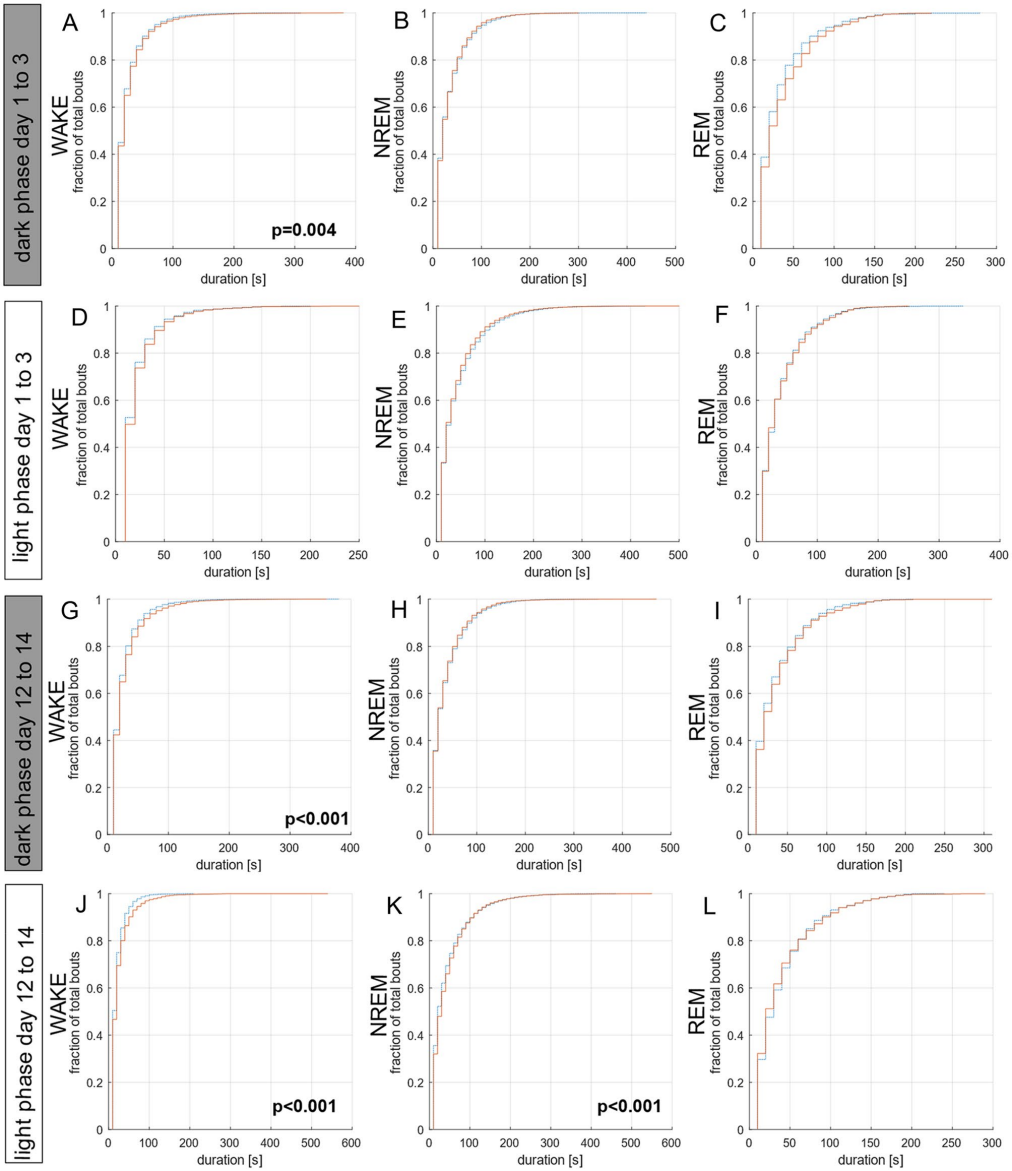
Bout length distribution of the vigilance states of the nontethered (broken blue lines, n = 6) and tethered (solid red lines, n = 7) animals pooled for dark phase and light phase. (**A**–**C**) Bout lengths of the dark phase at the beginning (first 3 days) of the experimental observation period for WAKE (**A**), NREM sleep (**B**) and REM sleep (**C**). Differences in the bout lengths for WAKE were detected (p = 0.004). (**D**–**F**) Bout lengths of the light phase at the beginning (first 3 days) of the experimental observation period for WAKE (**D**), NREM sleep (**E**) and REM sleep (**F**). (**G**–**I**) Bout lengths of the dark phase at the end (last 3 days) of the experimental observation period for WAKE (**G**), NREM sleep (**H**) and REM sleep (**I**). Differences in the bout lengths for WAKE were detected (p < 0.001). (**J**–**L**) Bout lengths of the light phase at the end (last 3 days) of the experimental observation period for WAKE (**J**), NREM sleep (**K**) and REM sleep (**L**). Differences in the bout lengths for WAKE (p < 0.001) and NREM (p < 0.001) were detected. Differences between the groups were tested using the Kolmogorov–Smirnov test and cumulative probability plots.

The cumulative probability of bout lengths was analyzed separately for the light phase and for the dark phase on day 1, at the beginning (first 3 days) and at the end (final 3 days) of the observation period in the glass monitoring cages (Fig. 4, Supplementary Fig. S6). We identified group differences in the bout lengths of NREM sleep in the dark phase on day 1 (p = 0.035; Supplementary Fig. S6) and in the light phase at the end (last 3 days) of the experimental observation period (p < 0.001; Fig. 4K). The more shallow slopes in the cumulative probability plots indicate that tethered animals exhibit a higher probability of longer NREM sleep bouts than nontethered animals.

An impact of tethering became also evident in the bout lengths of WAKE both in the dark phase at the beginning (p = 0.004; first 3 days) and the end (p < 0.001; last 3 days) of the experimental observation period (Fig. 4A,G) and in the light phase at the end (last 3 days; p < 0.001) of the experimental observation period (Fig. 4J). With a more shallow slope of the cumulative probability plots tethered animals appear to have a higher probability of longer WAKE bouts than nontethered animals.

Considering the differences in bout lengths of WAKE, we additionally analyzed the bout lengths for AWAKE and QWAKE, separately. Differences were identified in the bout lengths of QWAKE in the dark phase on day 1 (p < 0.001; Supplementary Fig. S6) reflected by more shallow slope of the respective cumulative probability curve of tethered versus nontethered animals indicating a higher probability of longer QWAKE bouts.

**Figure 5:**
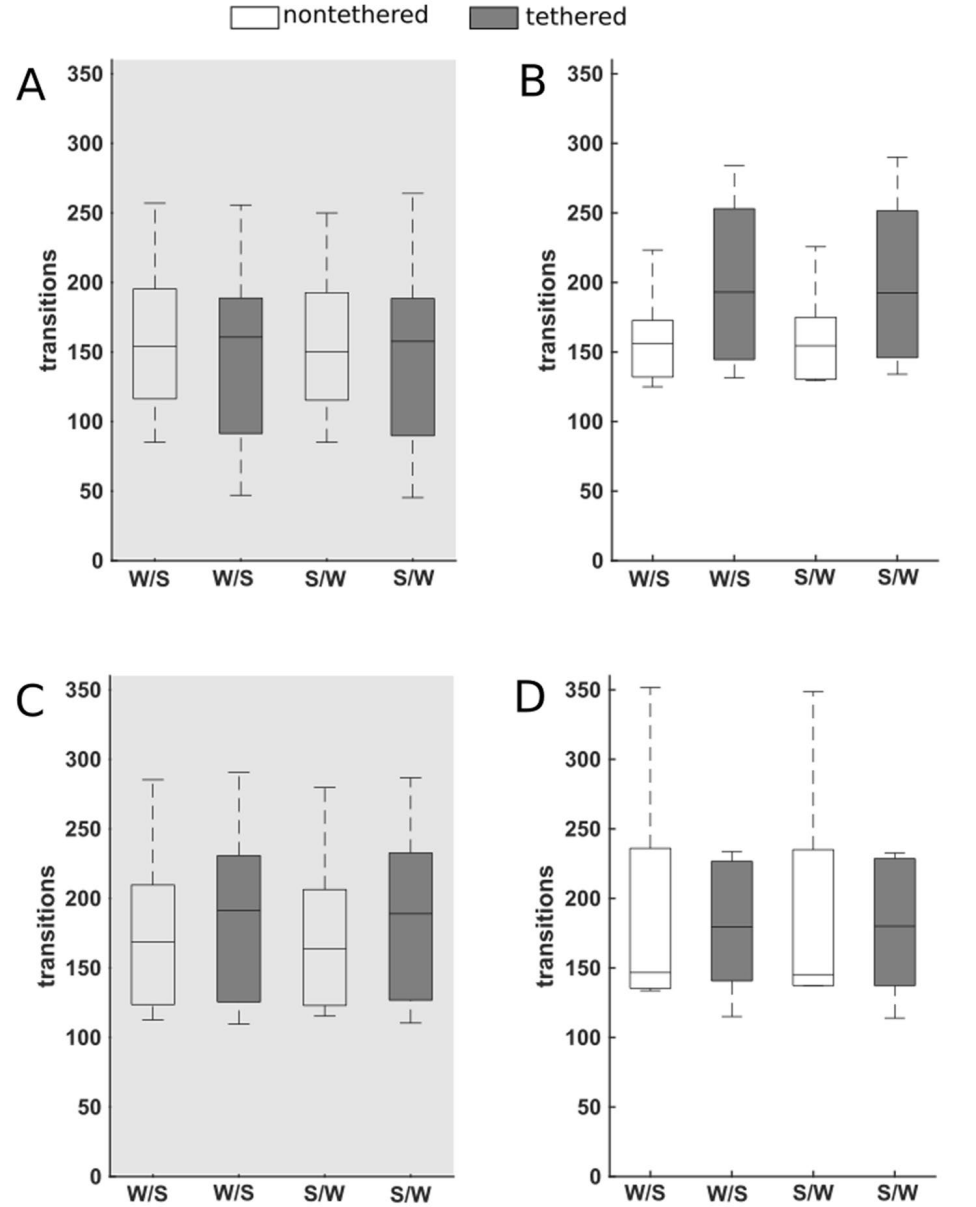
Transitions between WAKE and SLEEP of the nontethered (n = 6) and tethered (n = 7) animals. W/S = Transitions from WAKE to SLEEP. S/W = Transitions from SLEEP to WAKE. (**A**,**B**) Averaged values for the first 3 days of the experimental observation period (early) for the dark phase (**A**) and light phase (**B**). There was no significant difference between the nontethered and tethered animals in (**A**) the dark phase and in (**B**) the light phase. (**C**,**D**) Averaged values for the last 3 days of the experimental observation period (late) for the dark phase (**C**) and light phase (**D**). There was no significant difference between the nontethered and tethered animals in (**C**) the dark phase and in (**D**) the light phase. Box-plots show minimum to maximum values with median. Differences between the groups were tested using the Mann–Whitney-U test.

The analysis of the number of transitions between the vigilance states in the dark and light phase of day 1, at the beginning (first 3 days), and at the end (last 3 days) of the observation period in the glass monitoring cages did not confirm group differences (Fig. 5, Supplementary Figs. S7, S8, S9).

### Quantitative EEG analysis slow wave activity power

**Figure 6:**
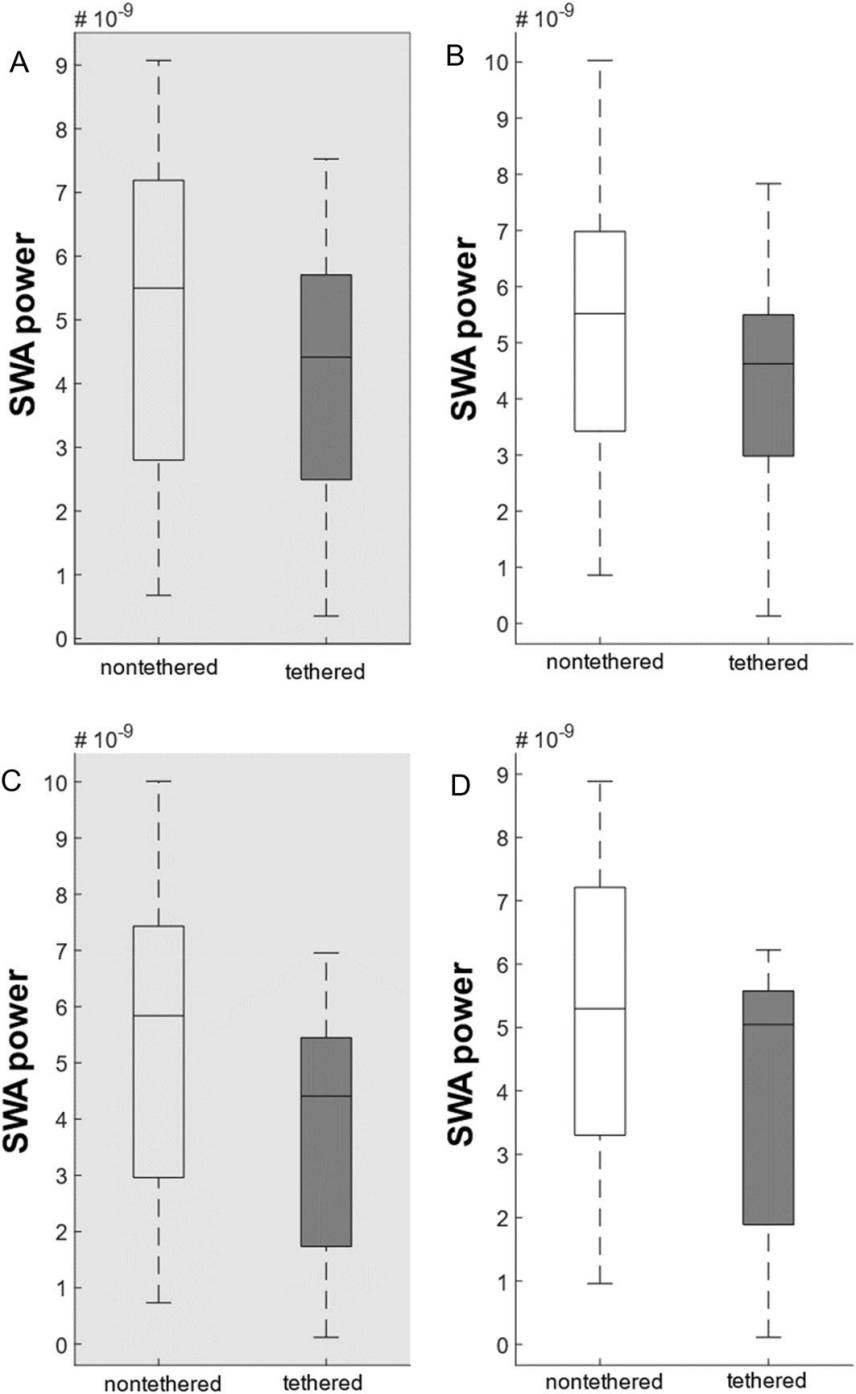
SWA power of the nontethered (n = 6) and tethered (n = 7) animals for each 10-s episode that was scored NREM. (**A**,**B**) Averaged values for the first 3 days of the experimental observation period (early) for the dark phase (**A**) and light phase (**B**). There was no significant difference between the nontethered and tethered animals in the dark phase (**A**) and in the light phase (**B**). (**C**,**D**) Averaged values for the last 3 days of the experimental observation period (late) for the dark phase (**C**) and light phase (**D**). There was no significant difference between the nontethered and tethered animals in the dark phase (**C**) and in the light phase (**D**). Box-plots show minimum to maximum values with median. Differences between the groups were tested using the Mann–Whitney-U test.

SWA power was analyzed separately for the light and dark phase on day 1, at the beginning (first 3 days), and at the end (last 3 days) of the observation period in the glass monitoring cages. This analysis did not identify any relevant differences in SWA power between groups (Fig. 6, Supplementary Fig. S10).

### Impact of the tethering cable on nest building and on saccharin preference

**Figure 7:**
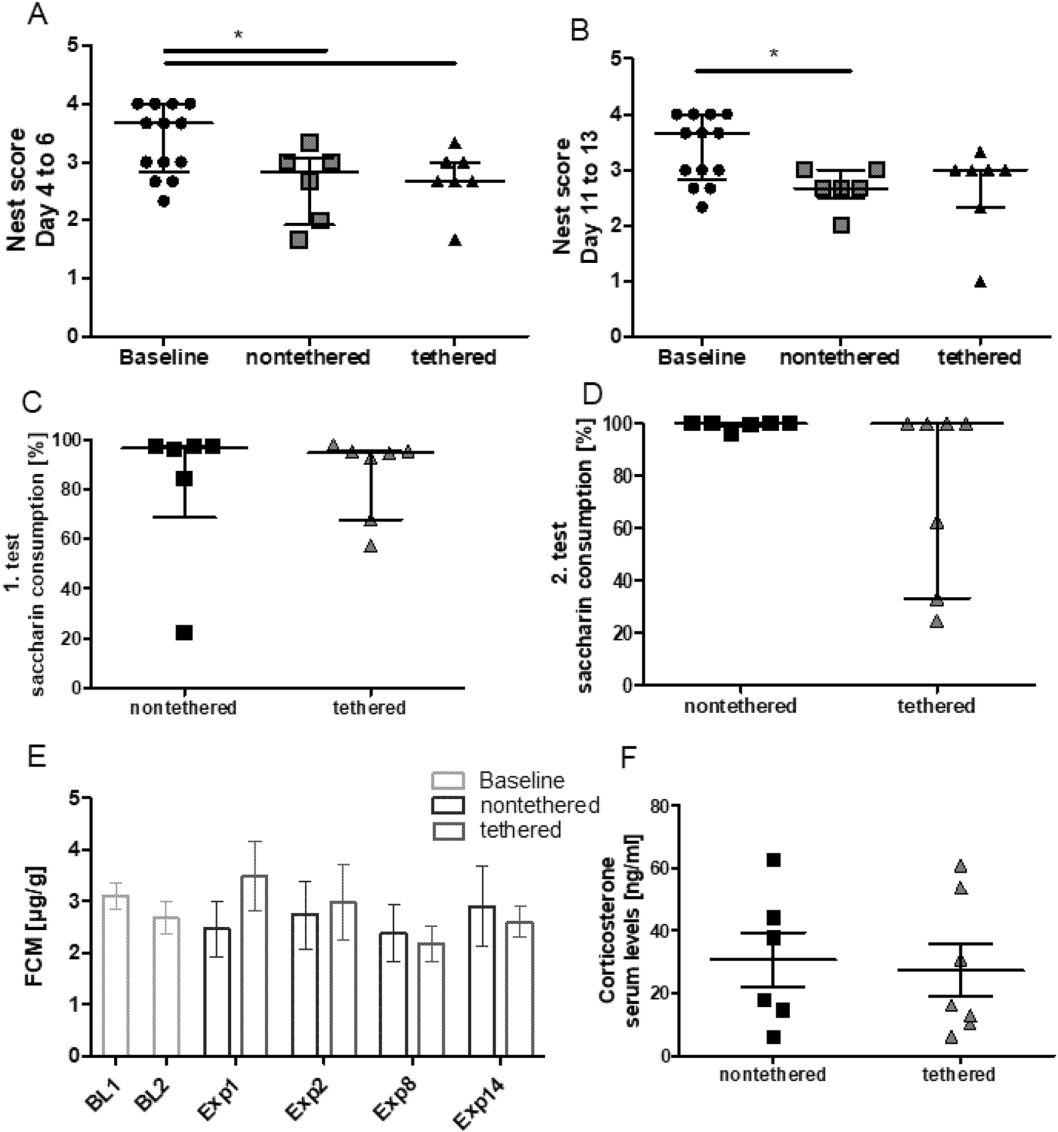
Nest building, anhedonia‐associated behavior, fecal corticosterone metabolite (FCM) levels and serum corticosterone levels for the nontethered (n = 6) and tethered (n = 7) animals. Baseline data: n = 13 animals. (**A**,**B**) Averaged nest building scores for day 4 to 6 (**A**) and day 11 to 13 (**B**). No significant differences between the nontethered and tethered animals were detected. (**C**,**D**) Anhedonia‐associated behavior for the first week (**C**) and the second week (**D**). No significant differences between the nontethered and tethered animals were detected. (**A**–**D**) Data are presented as median (interquartile range). Differences between the groups were tested using the Mann–Whitney-U test. An asterisk indicates a significant difference. (**E**) Fecal corticosterone metabolite (FCM) levels for Baseline 1 (BL1), Baseline 2 (BL2), Experimental day 1 (Exp1), Experimental day 2 (Exp), Experimental day 8 (Exp8) and Experimental day 14 (Exp14). No significant differences were detected. Data are presented as mean ± SEM. Differences between the groups were tested using a two-way ANOVA with factors “nontethered/tethered” and “days”, followed by a post-hoc Bonferroni multiple comparison test. (**F**) No significant differences between the nontethered and tethered animals were detected for the serum corticosterone levels. Data are presented as mean ± SEM. Differences were tested using an unpaired t-test (two-tailed).

Nest building activity has been suggested as an indicator for wellbeing in laboratory rodents^17,27^. Previous studies from our institute demonstrated that nest complexity scores stabilize and plateau 4 days after providing new nest material and decrease again at day seven^17,18^. Therefore, nest complexity was evaluated from day four to six after providing new nest material. Scoring of nest complexity during the observation period in the monitoring cages did not reveal significant group differences (Fig. 7). In both weeks a progressive increase in nest scores was evident in both groups of animals with and without connection to a cable. Maximum scores in nontethered and tethered rats were comparable reaching 3 in both groups. Median scores towards the end of the first week (Fig. 7A) and towards the end of the second week (Fig. 7B) were in a comparable range in nontethered and tethered rats, respectively. The assessment of nest complexity within groups during the observation period revealed no significant differences (Supplementary Fig. S11).

Saccharin preference was assessed twice during the phase in the glass monitoring cages. Based on data from days with exposure to two identical water bottles we were able to exclude a side preference as a potential bias. Continuous connection to a recording cable did not seem to affect the relative amount of saccharin consumed during the first (Fig. 7C) and second week (Fig. 7D) of exposure to the experimental condition in comparison to the nontethered group. Intra-group comparison revealed a significant increase in saccharin consumption (p = 0.031) in the nontethered animals between week 1 and 2, while the tethered animals showed no significant difference (Supplementary Fig. S11). Considering individual data, it was however interesting to note during the 2nd week that the results for nontethered rats were rather homogenous, whereas a high level of variance with rather low saccharin consumption in three tethered rats was evident.

### Impact of the tethering cable on corticosterone and its metabolites

Analysis of FCM offers opportunities for an assessment of adrenal gland activity without invasive sampling procedures. Three and a half week following surgery, i.e. before transfer of the rats to the glass monitoring cages, fecal corticosterone metabolites did not differ significantly from baseline levels prior to surgery (Fig. 7E). Transfer to the monitoring cage did not significantly influence the concentration of fecal corticosterone metabolites in comparison to baseline levels and to levels prior to the transfer (Fig. 7E). Moreover, no group differences were evident between animals with cable and without cable during the phase in the monitoring cages (Supplementary Fig. S12). Fecal corticosterone metabolites did not differ significantly between these groups at day 1, 2, 8, and 14 during this experimental phase (Fig. 7E). For the within-group comparison, no significant difference was found between time points in both groups (Supplementary Fig. S11).

Further supporting the lack of group differences in hypothalamic–pituitary–adrenal gland axis activity, serum corticosterone analyzed at the end of the experiment proved to be in the same range in animals with and without a 2-week cable connection (Fig. 7F).

### Calculation of the AUC for sleep/wake transitions, SWA power, behavioral and biochemial parameter

AUC values (orange, Supplementary Fig. S13) and 10,000-fold bootstrapped 95% confidence intervals completing the statistical comparisons presented in Figs. 5, 6, 7. None of the AUC values, except for the sleep–wake transition in the light phase, were > 0.7 or < 0.3. This means that the strength of the effect the different recording techniques had on the observed variable was poor at most (Supplementary Fig. S13, Supplementary Table S1).

### Impact of the tethering cable on activity counts

**Figure 8:**
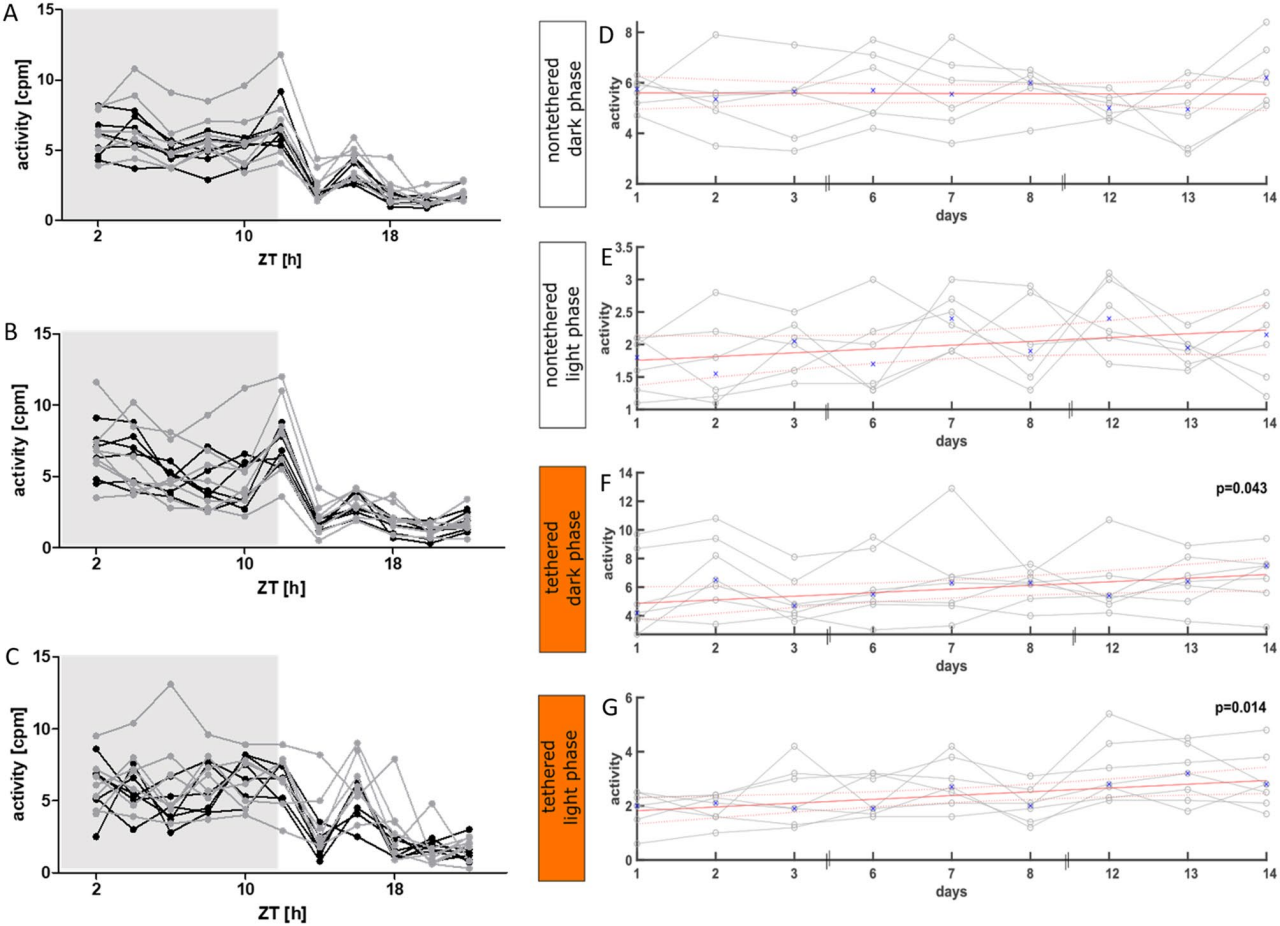
Activity analysis of the nontethered (n = 6) and tethered (n = 7) animals. (**A**–**C**) Activity counts of the nontethered (black) and tethered (gray) animals for nonoverlapping 2 h observation episodes throughout the 22 h. Lights were turned on after 12 h. Gray = dark phase, white = light phase. The x-axis represents Zeitgeber time (ZT). cpm = counts per minute. (**A**) Averaged values for all 9 days of the experimental observation period. The analysis detected no significant difference between the nontethered (black) and tethered (gray) animals at any time point. (**B**) Averaged values for the first 3 days (early) of the experimental observation period. The analysis detected no significant difference between the nontethered (black) and tethered (gray) animals at any time point. (**C**) Averaged values for the last 3 days (late) of the experimental observation period. The analysis detected no significant difference between the nontethered (black) and tethered (gray) animals at any time point. Data are plotted individually for each animal. Differences between the groups were tested using a two-way ANOVA with factors “nontethered/tethered” and “hours”, followed by a post-hoc Bonferroni multiple comparison test. (**D**–**G**) Linear regression model of activity counts of the nontethered and tethered animals throughout the experimental observation phase. For each animal, the average per day pooled for the dark phase and light phase was calculated. The x-axis represents the days. (**D**,**E**) Linear regression model of activity counts of the nontethered animals for the dark phase (**D**) and light phase (**E**). The activity level was not significantly different over the time course of the observation period. (**F**,**G**) Linear regression model of activity counts of the tethered animals for the dark phase (**F**) and light phase (**G**). A significant increase in activity counts was analyzed for the dark phase (p = 0.043) and the light phase (p = 0.014). Data are plotted individually for each animal.

The activity counts for nonoverlapping, consecutive 2 h observation episodes throughout the 22 h observation period was analyzed considering all experimental observation days. In addition, we separately analyzed data from day 1, the first three, and the final 3 days of the observation period, at the beginning, and at the end of the phase in the glass monitoring cages. The activity counts were not significantly different between animals with and without a cable connection during baseline recording and at any time point during the experimental observation phase (Fig. 8A–C, Supplementary Fig. S14).

For the nontethered animals, the average number of activity counts was not significantly different between the beginning and end of the observation period in both the dark phase and the light phase (Fig. 8D,E). For tethered animals, a significant increase in the average number of activity counts during both the dark phase (p = 0.043) and the light phase (p = 0.014) was evident throughout the observation period (Fig. 8F,G).

## Discussion

It has been repeatedly stated that the replacement of tethered by telemetric recordings can increase data quality and can have relevant effects on the animal’s wellbeing and experiment-associated distress^2–4,6,28^. Based on previous findings, we aimed to analyze the impact of tethered recordings in female rats in more detail with a focus on vigilance cycling and activity and sleep patterns. In addition, the stress hormone corticosterone and its metabolites as well as home cage behavior were analyzed.

Nest building activity is discussed as a behavioral parameter, which can provide valuable information about the affective and clinical state of an animal^27^. Reduced nest quality has been reported as a consequence of research-associated distress and pain in different animal models and as a consequence of various experimental interventions (e.g. Refs.^27,29–31^). Both the comparison between tethered and non-tethered animals and the within-group comparison during the experimental observation period did not reveal any differences in nest building activity.

This finding seems to be in line with the lack of differences in saccharin preference. The loss or reduction of a preference for sweet solutions has been interpreted as anhedonia-associated behavior in rodents, which may reflect depression-associated behavioral patterns in humans^32^. We have recently started to validate saccharin preference as a parameter for evidence-based severity assessment in mice and rats. In rats, principal component analysis of comprehensive data sets with findings from a long-list of behavioral, physiological, and biochemical parameters analyzed in different epilepsy models identified saccharin preference among the top parameters indicating differences in the affective state of experimental and naïve control animals^33^. More recently, we provided first confirmation that saccharin-preference may also serve as a severity assessment parameter in respective models in mice^34^. Taken together, the analysis of nest quality and saccharin preference argues against a pronounced impact of tethered recordings on the affective state. However, saccharin consumption and preference showed increased interindividual variance in tethered rats only. This might suggest individual differences in the ability to adjust to tethering and cope with the associated distress. While daily inspections should be considered a matter of course, we nevertheless want to emphasize that careful daily controls of individual animals with tethered recordings including a detailed analysis of behavioral patterns are of utmost relevance.

Despite all technical efforts to limit the traction forces on the tethering cable, respective influences on the animal cannot be entirely avoided. Therefore, we have addressed the hypothesis that tethered rats exhibit alterations in vigilance cycling and sleep quality due to traction forces affecting their movement, locomotion, resting, and sleep patterns.

The level and potential influence of the semi-restraint situation described by different authors^2–6^ will likely depend on the detailed technical setup. In the present study, we have used a tethering system with a long cable and swivel without a counterbalance allowing movement throughout the entire monitoring cage. The study assessed the influence of a 2-week exposure to this tethering system with continuous cable connection in tethered rats.

Combined electroencephalographic and electromyographic recordings allow to distinguish four different vigilance states comprising active awake, quiet awake, NREM sleep and REM sleep.

An analysis of the cumulative time in the different vigilance states did not reveal group differences. Thus, neither the total sleep time nor the total time with NREM or REM sleep was affected by tethering. These data indicate a lack of influence of the cable connection on total sleep time and quality concerning the relative ratio of NREM and REM sleep, though one needs to consider that age-dependent mechanisms of sleep homeostasis have been described^35^, which may counterbalance disturbed sleep patterns so that a compensation may occur over the analyzed time frame. Thus, we have additionally analyzed cycling between vigilance states.

Unlike humans, rats and other rodents exhibit frequent cycling between different vigilance states during the both phases of the day, i.e. the dark and light phase^36^. While the dark phase is characterized by a dominance of wake phases, a higher total sleep time characterizes the light phase^36^. Aiming to analyze a possible disturbance of activity and wake/sleep patterns with an increased sleep fragmentation triggered by an experimental procedure, it is therefore of particular relevance to assess the mean length of the different wake and sleep phases and the number of transitions between these phases. Data from the first day, the beginning of the observation phase (first 3 days) and from the end of the observation phase (last 3 days) were analyzed separately in order to obtain information about a possible habituation to the tethered condition. Surprisingly, during the dark phase of day 1 NREM sleep bouts tended to be longer in tethered rats. Moreover, WAKE bout lengths proved to be longer when analyzing data from the dark phase of the first day or the first 3 days. Thereby a more detailed analysis revealed an increased duration of quiet awake bouts during the dark phase of the first monitoring day. These findings argue against a traction force mediated arousal during sleep phases and rather indicate an influence of the tethered state on vigilance cycling with a trend towards a prolonged lingering in selected vigilance phases. On the other hand, it might be that traction forces delay sleep induction, which is then in line with sleep homeostatic responses compensated by longer NREM sleep bouts. Towards the end of the observation phase minor group differences were still evident with tethered rats showing a trend towards longer NREM bouts during the light phase and longer WAKE bouts during the dark and light phase.

While these findings may point to a slight impact of tethering on vigilance cycling, a respective influence is not confirmed by alterations in the number of transitions between the respective vigilance states.

In rats and other rodents, it was clearly shown that the duration of preceding wakefulness is one of the main factors influencing subsequent overall sleep duration and especially sleep quality^15,16^. In concordance with our results on vigilance state differences, we found no significant differences in SWA between tethered and nontethered rats, suggesting that prior wakefulness is qualitatively and quantitatively very similar in both groups.

Interestingly, we still noticed a small, although not significant overall trend of higher SWA power in nontethered rats in comparison to tethered animals. In rats, running wheel access tends to lead to more, although not significant SWS^37^ while SWA itself increases for example in humans^38^ and rats^39^ after physical exercise. It may be that the tethered rats showed slightly less motor activity (equally to physical exercise) during the beginning of the experimental observation phase in comparison to the nontethered group resulting in slightly, non-significantly lower slow wave power.

Sleep deprivation^16^ also increases SWA in rats (and other species). Assuming that an increased amount of quiet wakefulness/increased bout length of quiet wakefulness as shown in tethered rats (Suppl. Fig. S4H) acts similarly on sleep homeostasis as sleep deprivation one could argue that tethered rats should have increased SWA. Whether the significant differences in bout length are relevant for subtle differences in SWA is as speculative as the involvement of increased physical activity mentioned above. Based on these weak arguments and the non-significant differences in SWA between both experimental groups, we assume similar quality of SWS in tethered and nontethered animals, at least in our experiments.

Taken together considering the lack of an impact on cumulative time in vigilance states and on number of sleep/wake and wake/sleep transitions as well as the rather limited changes in the lengths of NREM sleep and WAKE and the similar quality of SWS, the findings from rats with a cable connection rather argue against a profound detrimental impact on sleep patterns and quality. In this context it is important to note that the comparable cumulative time in the different vigilance states confirms that, even considering the slight changes in bout lengths, homeostatic mechanisms are by and large not compromised so that a compensation is possible.

Interindividual differences in the response to factors that may affect vigilance cycling, and sleep patterns have previously been described^40–43^. While our study argues against relevant group differences, one tethered rat was standing out with an extremely low rate of sleep episodes during the dark and light phase of day 1. This finding suggests that a pronounced effect of tethered recordings on sleep patterns may be possible in individual animals. This conclusion, and analysis of the possible reasons for it, will require follow-up studies in larger animal groups.

Analysis of fecal corticosterone metabolites allows to repeatedly assess a potential impact of experimental procedures on stress hormone levels without invasive sampling techniques that can bias the outcome^44^. It has previously been demonstrated that for example sleep deprivation induced by motorized activity wheels can increase fecal corticosterone metabolites in rats^45^. Thus, the lack of any relevant influence on fecal corticosterone metabolites is in line with our findings that sleep patterns and quality were not affected by tethering.

In this context, it is of interest that corticosterone serum concentrations did not differ between groups. As handling can trigger an acute rise in naïve animals^18,44^, these data also suggest that rats with and without a continuous cable do not differ in their response to handling. Thus, these findings taken together with the behavioral data do not confirm any relevant difference in anxiety or stress levels.

In mice exposed to chronic stress, a reduction in distance moved on the running wheel was analyzed, with the greatest effect observed at the beginning of the experiment^46^. The results of our activity count analysis also confirmed an effect on distance moved after exposure to the cable. We found a significant increase in the average activity level from the beginning to the end of the observation period in the tethered animals. However, it is interesting to note that the average proportion of epochs in AWake did not increase significantly during the observation phase. Thus, although we did not find an effect of the tethering cable on the proportion in AWake, the level of activity (i.e., speed and distance traveled) was influenced by the tethering cable. However, it remains unclear whether habituation to the movement restriction caused by the cable, habituation to the stress caused by the cable, or a combination of both factors led to an increase in activity level and distance traveled. We would like to emphasize that when the activity was recorded, a count number was generated depending on the distance traveled and the speed. Thus, the exact distance traveled and speed were not determined. Equipping the animals with an accelerometer would allow such an exact measurement of the distance and speed traveled. Therefore, it would be of interest to investigate the activity in more detail in follow-up studies.

In summary, data sets from rats with and without cable connection for 2 weeks argue against a pronounced impact on the animal’s affective state, vigilance cycling, sleep quality, and hypothalamic–pituitary–adrenal gland axis activity. Concerning locomotion and activity the findings provide evidence for an impact of a prolonged tethered condition on activity levels. However, the lack of significant group differences argues against a major influence.

In this context, it needs to be considered that it was not possible to integrate a meaningful control group such as a naïve group due to the fact that the main readout parameters required a transmitter implant. Thus, the findings only allow conclusion about the comparison between the tethered and non-tethered state. The data should not be misinterpreted in a sense that experimental recording approaches including the surgical procedures do not have an influence on animal welfare.

Both experimental approaches, tethered and telemetric recordings, required a surgical intervention for the electrode implants. In this context, the need of an additional transmitter implant requires attention for animals prepared for telemetry. While previous data from rats and mice argued against pronounced long-term effects of electrode and transmitter implants on various sensitive behavioral and biochemical severity assessment parameters, data from the present and earlier studies demonstrated the influence on the animal’s wellbeing with a transient increase in RGSs, general clinical and Irwin scores.

It is also important to consider the relevance of various factors including housing conditions, handling procedures, habituation phases, age, sex, and genetic background that can affect the ability of laboratory animals to cope with stress associated with experimental conditions and interventions. In this context, the influence of age and estrous cycle on homeostatic sleep regulation needs to be taken into account^35,47–49^. Thus, the generalizability of our findings needs to be further assessed and a multicenter study would be of interest. In these studies the influence of sex should also be analyzed by direct comparison between female and male animals.

In addition, recordings are often conducted in models of neurological disorders^2,6^. Respective models are often characterized per se by changes in sleep patterns and homeostatic responses modulating sleep (e.g. Refs.^25,50,51^), which may also affect the ability of animals to adjust to experimental conditions.

Telemetric studies are also frequently conducted in mice, sometimes even during early postnatal development^3,5,6^ and in aged animals, were sleep is generally more fragmented. Therefore, the impact of recording conditions must be determined for each specific experimental study and may differ considering the relative impact of cable traction forces on smaller or older animals, and with regards to size ratios between transmitters and animals.

In conclusion, the comparison of behavioral and sleep patterns in rats with and without cable connection did not identify major group differences. Our findings indicate that the refinement effect of telemetric recordings replacing tethered recording might be relatively minor or might not exist in rats. In this context, it needs to be considered that severity assessment always requires a case-by-case analysis.

## Supplementary Information

Supplementary Information.

## Data Availability

The datasets generated or analyzed during this study are available on the DFG data repository. Link: https://for.severity-assessment.de/. The data on the statistical analysis of the sleep patterns are available from M.K. at TUM.
