# Supplementary material for: The impact of tethered recording techniques on activity and sleep patterns in rats
Source: Sci Rep. 2022 Feb 24;12:3179. doi: 10.1038/s41598-022-06307-3 (PMC8873297; doi:10.1038/s41598-022-06307-3)
Supplement: Supplementary file 1 — Supplementary Information. [file 41598_2022_6307_MOESM1_ESM.pdf]

SUPPLEMENTARY INFORMATION AND DATA

The impact of tethered recording techniques on activity and sleep patterns in rats

Katharina Aulehner[1], Jack Bray[2], Ines Koska[1], Claudia Pace[1], Rupert Palme[3], Matthias Kreuzer[4], Bettina Platt[2], Thomas Fenzl[4], Heidrun Potschka[1*]

1 Institute of Pharmacology, Toxicology, and Pharmacy, Ludwig-Maximilians-University, Munich, Germany

2 School of Medical Sciences, Institute of Medical Sciences, University of Aberdeen, Foresterhill, Aberdeen, Scotland, UK

3 Department of Biomedical Sciences, University of Veterinary Medicine, Vienna, Austria

4 Department of Anesthesiology and Intensive Care, School of Medicine, Technical University of Munich, 81675 Munich, Germany

*Corresponding author: Prof. Dr. Heidrun Potschka, Königinstr. 16, D-80539 München; potschka@pharmtox.vetmed.uni-muenchen.de

1. *Surgical procedure*

The surgeries were performed during the light phase (08:00 - 15:00) under aseptic conditions. General anesthesia was induced with 4% isoflurane (Isofluran CP®, Henry Schein Vet, Hamburg, Germany) and maintained at 2%. Perioperative pain management consisted of meloxicam s.c. (2 mg/kg, Metacam®, Boehringer Ingelheim, Germany, 30 min pre- and 24h, 48h, 72h post-surgery) and metamizole s.c. (100 mg/kg, Vetalgin®, Covetrus, Germany, during surgery). To surgically implant the telemetric transponder and electrodes, animals were placed in a stereotactic frame and the surgical sites were shaved prior to disinfection with iodine povidone (Braunol®, B.Braun, Melsungen, Germany). The local anesthetic bupivacaine (4 mg/kg, 0.5%, Jenapharm®, Mibe GmbH, Brehna, Germany) was injected subcutaneously into the site of transmitter implantation. Bupivacaine with epinephrine (4 mg/kg, 0.5% + 0.0005 %, Jenapharm®, Mibe GmbH, Brehna, Germany) was injected subcutaneously into the surgical site of electrode implantation in the skull. The telemetric transponder was implanted subcutaneously paramedian to the spine on the left side in the area over the thorax. The EMG electrodes were implanted into the neck muscle. The surface screw electrodes were implanted according to Jyoti et al., 2010[1] except that only two surface electrodes were used. The hippocampal coordinates were translated to rats according to Paxinos and Watson's rat brain atlas[2]. Both screw electrodes were implanted in drilled burr holes in the skull, one over the hippocampus (ap +3.9; lat +2.3) and the other as a reference over the cerebellum (Bregma ap +10.5). The screws were fixed onto the skull with a headcap made from dental cement (Paladur, Heraeus®, Hanau, Germany). The incisions were closed with single interrupted stitches using MonoPlus 5/0 (Smi AG, St. Vith, Belgium). To prevent postoperative infections, the animals were injected subcutaneously twice daily with marbofloxacin (Marbofloxacin; Marbocyl, 4 mg/kg, 1%, Covetrus, Hamburg, Germany) for eight days starting one day before surgery.

Supplementary Figure S1: Swivel-tether system

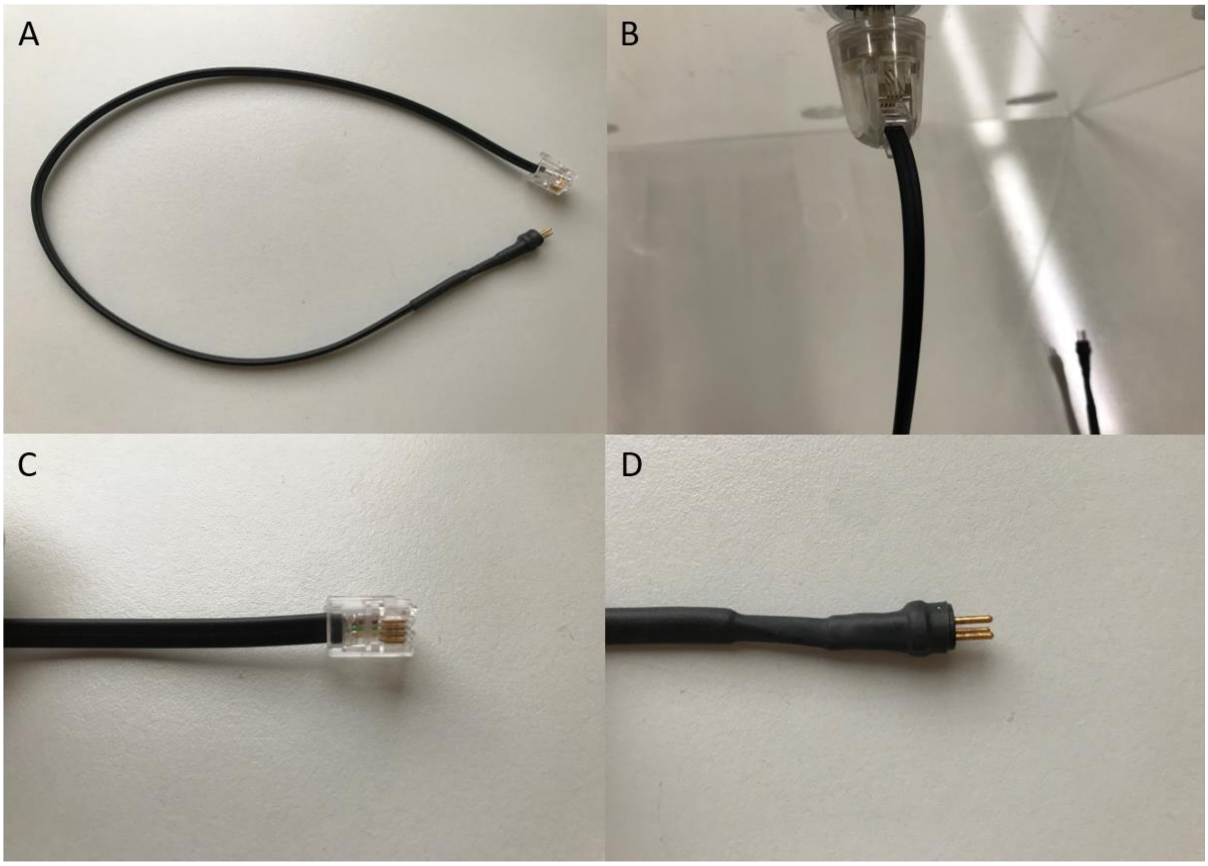

Cable (A) for the tethered group. Cable connected with the swivel-tether system (B). Distal (C) and proximal (D) end of the cable.

Supplementary Figure S2: Rat grimace scale, Irwin score and body weight

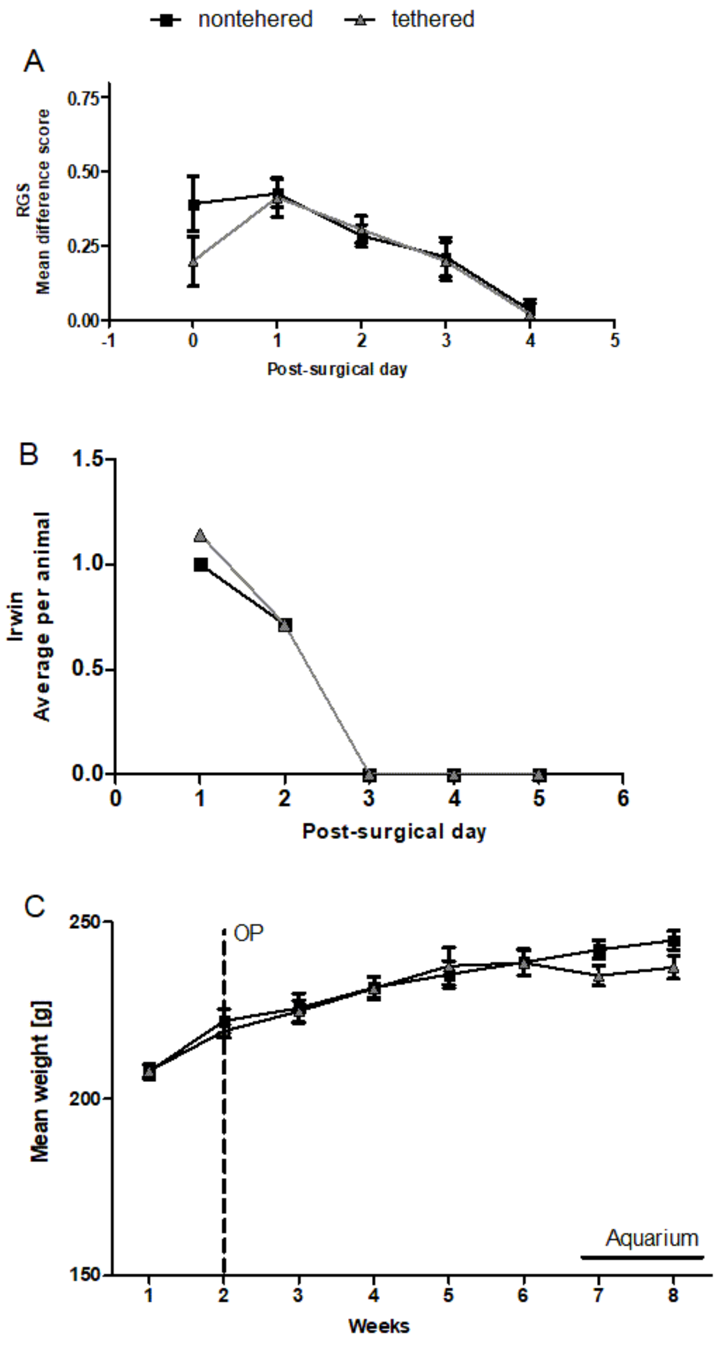

Rat grimace scale (A) assessed on post-surgical days 0 to 4 (n = 14). Irwin score (B) assessed on post-surgical days 1 to 5 (n = 14). Body weight (C) of the nontethered (n = 6) and tethered animals (n = 7) throughout the study from arrival to the end of the experimental observation period.

Supplementary Figure S3: Hypnogram Baseline Day 3

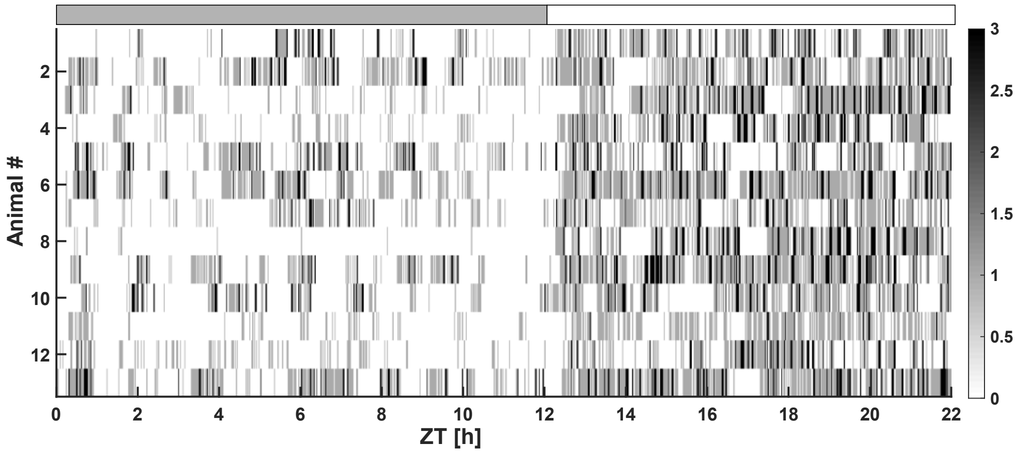

Hypnogram of 3rd day of baseline recording throughout the 22 h experimental observation period. Lights were turned on after 12 hours. The x-axis represents Zeitgeber time (ZT). The gray rectangle exhibits the dark phase and the white rectangle exhibits the light phase. White exhibits WAKE, light gray exhibits NREM sleep and dark gray exhibits REM sleep. #: individual animal number.

Supplementary Figure S4: Proportion of the vigilance states Baseline and Day1

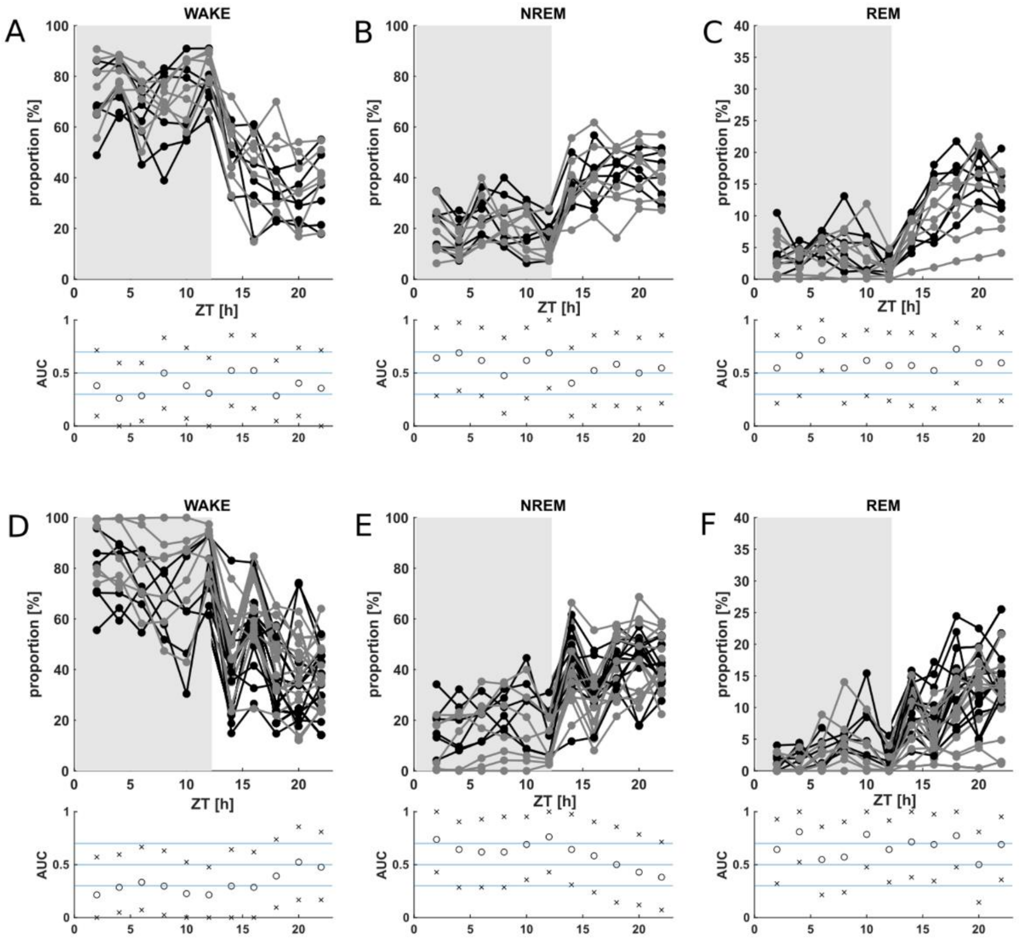

Proportion of the vigilance states of the nontethered (black, n = 6) and tethered (gray, n = 7) animals for non-overlapping 2 h observation episodes throughout the 22 h. Lights were turned on after 12 hours. The x-axis represents Zeitgeber time (ZT). Circles represent the calculated AUC. The limits of the 95% confidence intervals are represented by the letter x. NREM: NREM sleep, REM: REM sleep **A, B, C**: Averaged baseline days for WAKE (A), NREM sleep (B) and REM sleep (C). The AUC analysis detected no effect between the nontethered (black) and tethered (gray) animals. **D, E, F:** Averaged values on day 1 of the experimental observation period for WAKE (D), NREM sleep (E) and REM sleep (F). The AUC analysis detected no effect between the nontethered (black) and tethered (gray) animals. Data are plotted individually for each animal.

Supplementary Figure S5: Linear regression model of the vigilance states

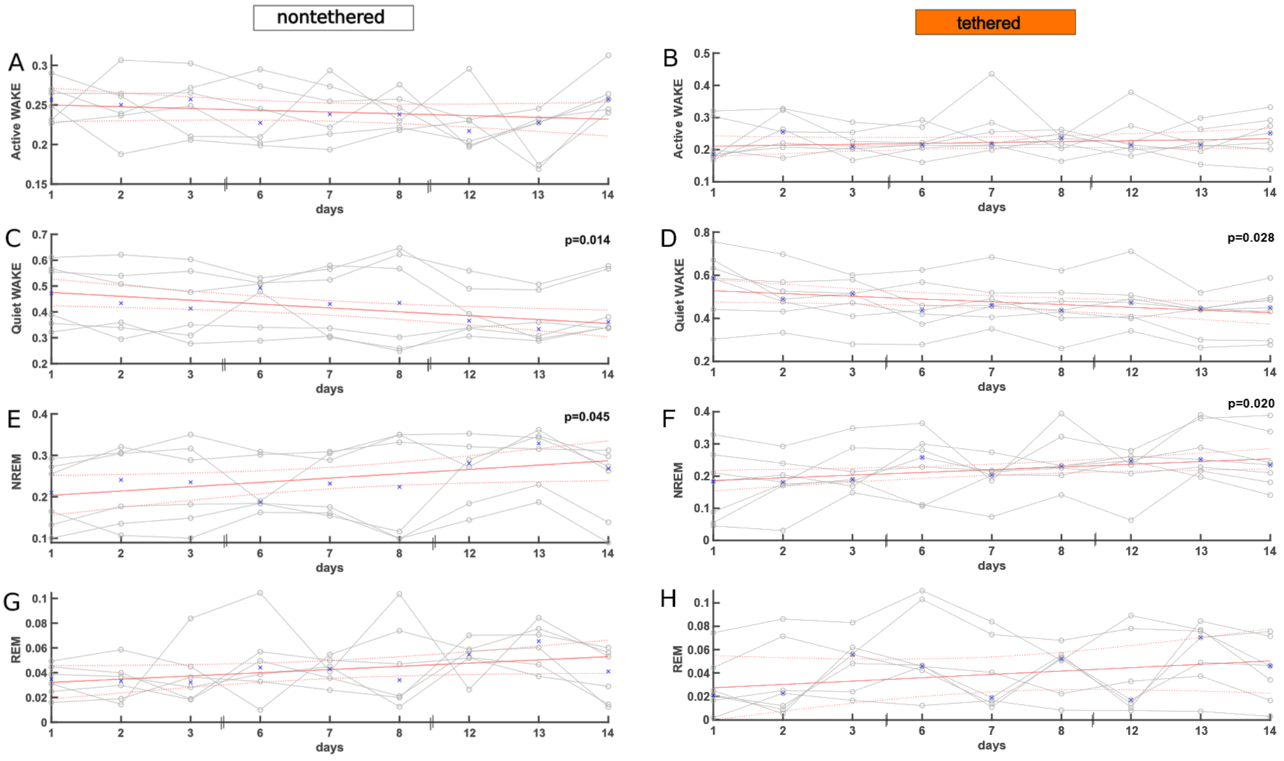

Linear regression model of the vigilance states of the nontethered (n = 6) and tethered (n = 7) animals throughout the experimental observation phase. For each animal, the average per day was calculated. The x-axis represents the days. NREM: NREM sleep, REM: REM sleep. **A, C, E, G**: Linear regression model for the nontethered animals for active WAKE (A), quiet WAKE (C), NREM sleep (E) and REM sleep (G). A significant decrease in quiet WAKE (p = 0.014) and a significant increase in NREM sleep (p = 0.045) from the early to the late experimental observation phase was detected. **B, D, F, H**: Linear regression model for the tethered animals for active WAKE (B), quiet WAKE (D), NREM sleep (F) and REM sleep (G). Data are plotted individually for each animal. A significant decrease in quiet WAKE (p = 0.028) and a significant increase in NREM sleep (p = 0.020) from the early to the late experimental observation phase was detected.

Supplementary Figure S6: Bout length distribution day 1 and AWAKE/QWAKE

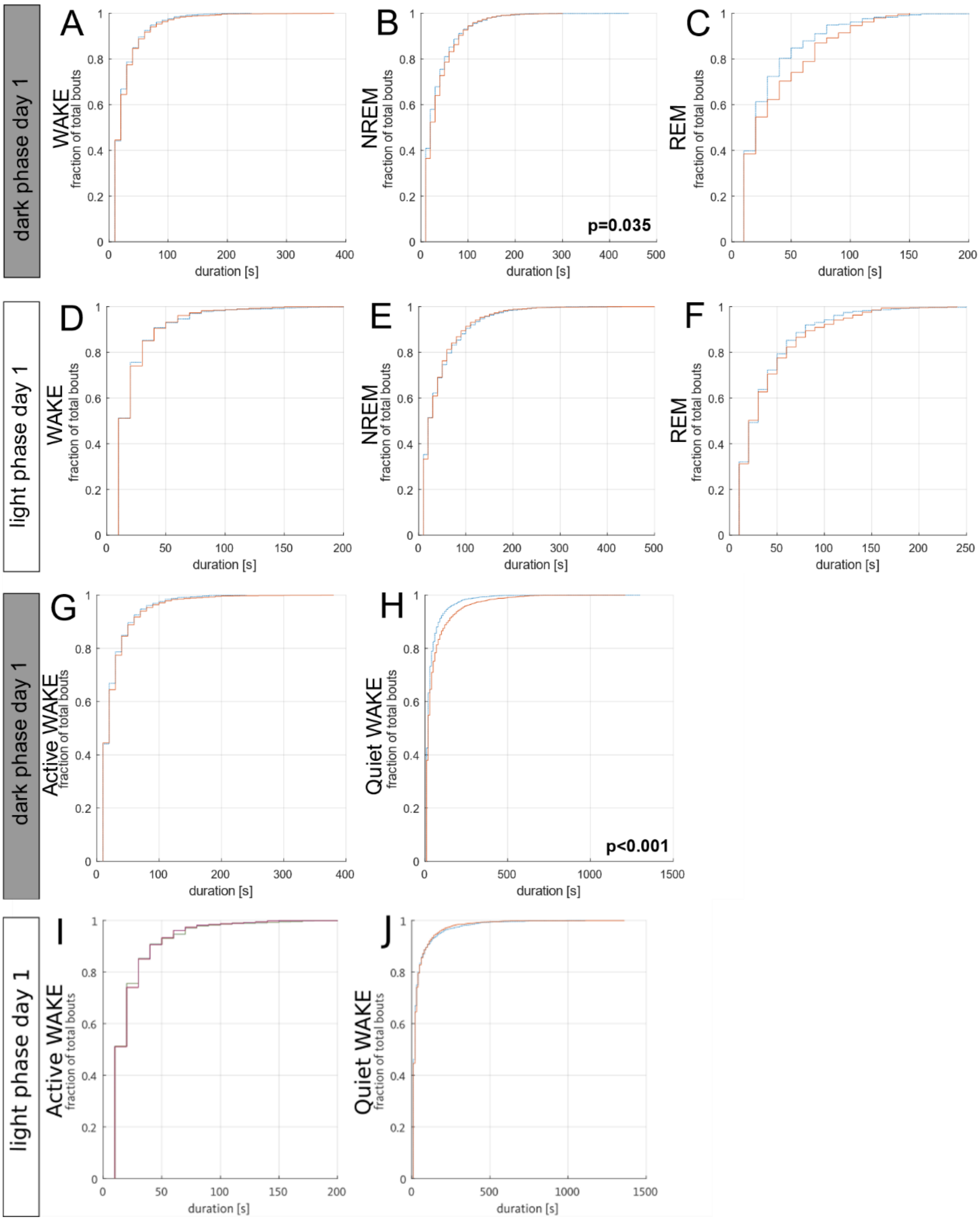

Bout length distribution of the vigilance states of the nontethered (broken blue lines, n = 6) and tethered (solid red lines, n = 7) animals pooled for dark phase and light phase. **A, B, C:** Bout lengths of the dark phase on day 1 of the experimental observation period for WAKE (A), NREM sleep (B) and REM sleep (C). Differences in the bout lengths for NREM were detected (p = 0.035). **D, E, F**: Bout lengths of the light phase on day 1 of the experimental observation

period for WAKE (D), NREM sleep (E) and REM sleep (F). **G, H**: Bout lengths of the dark phase on day 1 of the experimental observation period for active WAKE (G) and quiet WAKE (H). Differences in the bout lengths for quiet Wake were detected ($p < 0.001$). **I, J:** Bout lengths of the light phase on day 1 of the experimental observation period for active WAKE (I) and quiet WAKE (J). Differences between the groups were tested using the Kolmogorov-Smirnov test and cumulative probability plots.

Supplementary Figure S7: Transitions between NREM sleep and REM sleep

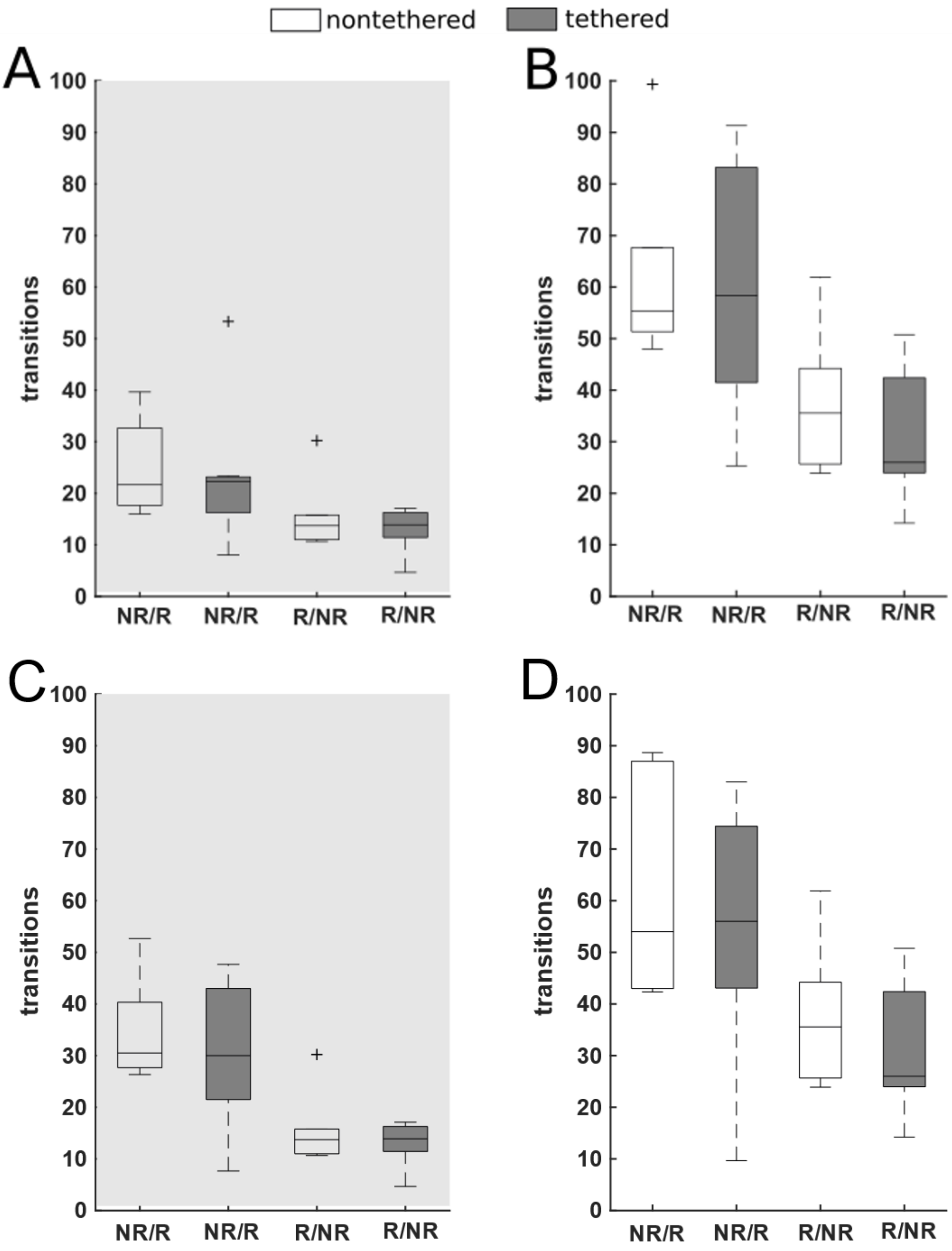

Transitions between NREM sleep and REM sleep of the nontethered and tethered animals. NR/R = Transitions from NREM sleep to REM sleep. R/NR = Transitions from REM sleep to NREM sleep. "+" exhibit outlier **A, B:** Averaged values for the **first three days** of the experimental observation period (early) for the dark phase (A) and light phase (B). There was no significant difference between the nontethered and tethered animals in (A) the dark phase

and in (B) the light phase. **C, D:** Averaged values for the **last three days** of the experimental observation period (late) for the dark phase (C) and light phase (D). There was no significant difference between the nontethered and tethered animals in (C) the dark phase and in (D) the light phase. Box-plots show minimum to maximum values with median. Differences were tested using the Mann-Whitney-U test.

Supplementary Figure S8: Transitions between QWAKE and AWAKE

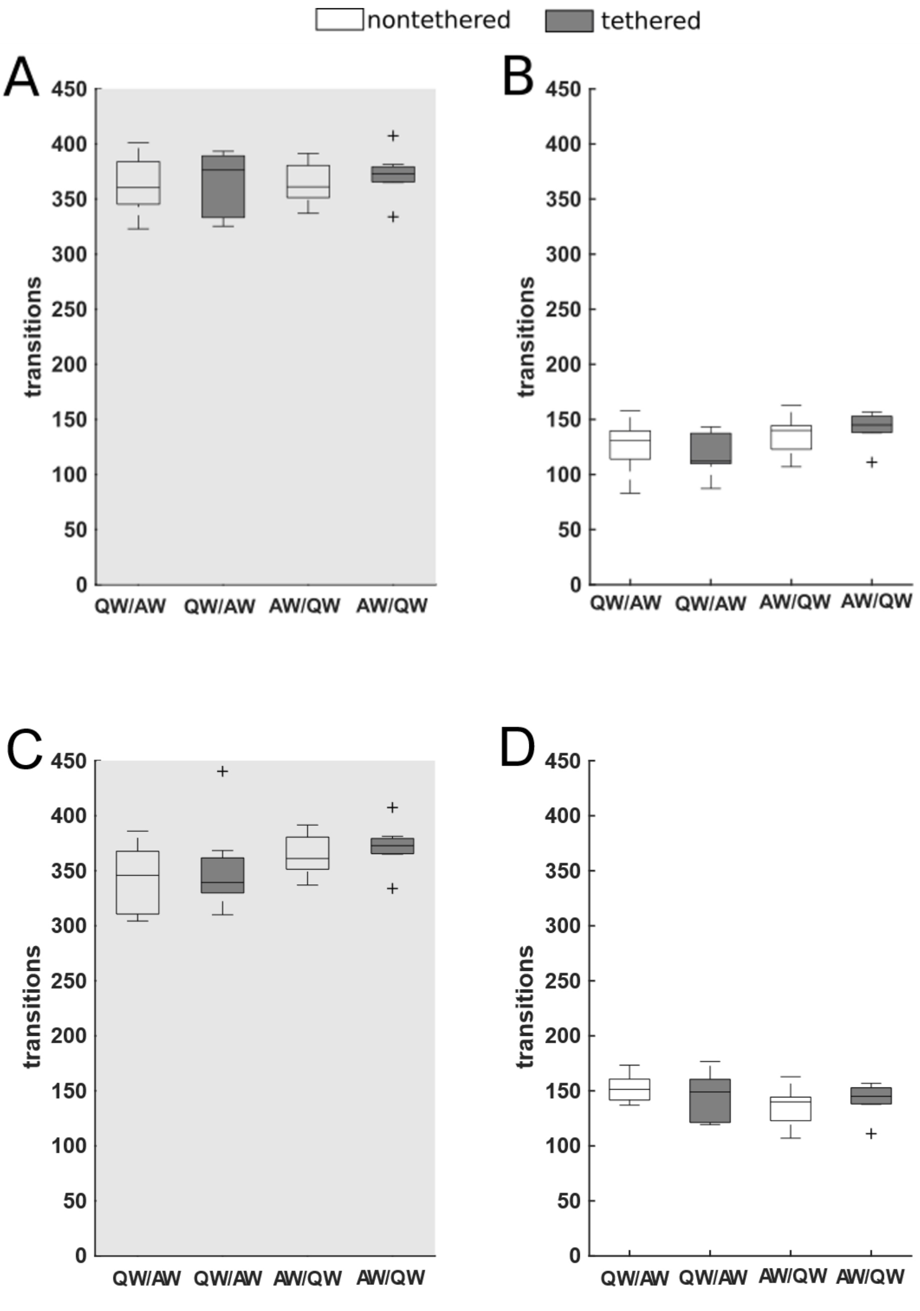

Transitions between quiet WAKE and active WAKE of the nontethered and tethered animals. QW/AW = Transitions from quiet WAKE to active WAKE. AW/QW = Transitions from active WAKE to quiet WAKE. "+" exhibit outlier **A, B:** Averaged values for the **first three days** of the experimental observation period (early) for the dark phase (A) and light phase (B). There was no significant difference between the nontethered and tethered animals in (A) the dark phase and in (B) the light phase. **C, D:** Averaged values for the **last three days** of the

experimental observation period (late) for the dark phase (C) and light phase (D). There was no significant difference between the nontethered and tethered animals in (C) the dark phase and in (D) the light phase. Box-plots show minimum to maximum values with median. Differences were tested using the Mann-Whitney-U test.

Supplementary Figure S9: Transitions between the vigilance states Day 1

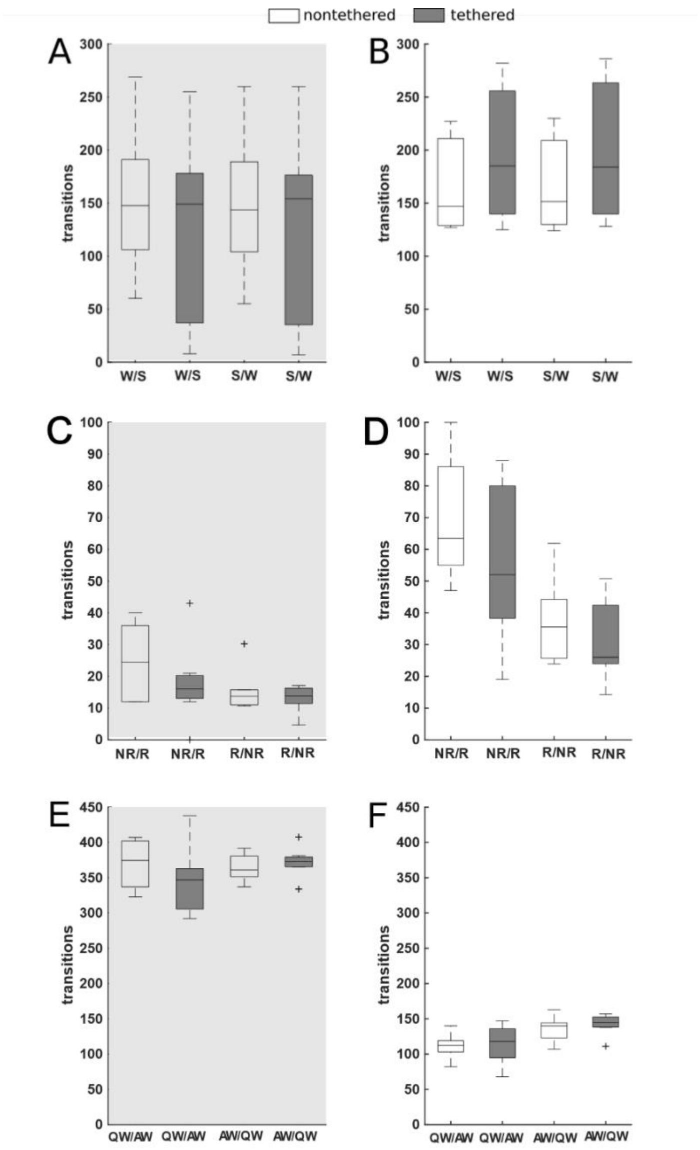

Transitions between the vigilance states **on the first day** of experimental observation period of the nontethered (n = 6) and tethered (n = 7) animals. W/S = Transitions from WAKE to SLEEP. S/W = Transitions from SLEEP to WAKE. NR/R = Transitions from NREM sleep to REM sleep. R/NR = Transitions from REM sleep to NREM sleep. QW/AW = Transitions from quiet WAKE to active WAKE. AW/QW = Transitions from active WAKE to quiet WAKE. "+" exhibit outlier **A, B:** Transitions between WAKE and SLEEP for the dark phase (A) and light phase (B). There was no significant difference between the nontethered and tethered animals in (A) the dark phase and in (B) the light phase. **C, D:** Transitions between NREM sleep and REM

sleep for the dark phase (C) and light phase (D). There was no significant difference between the nontethered and tethered animals in (C) the dark phase and in (D) the light phase. **E, F:** Transitions between quiet WAKE and active WAKE for the dark phase (E) and light phase (F). There was no significant difference between the nontethered and tethered animals in (E) the dark phase and in (F) the light phase. Box-plots show minimum to maximum values with median. Differences were tested using the Mann-Whitney-U test.

Supplementary Figure S10: SWA power day 1

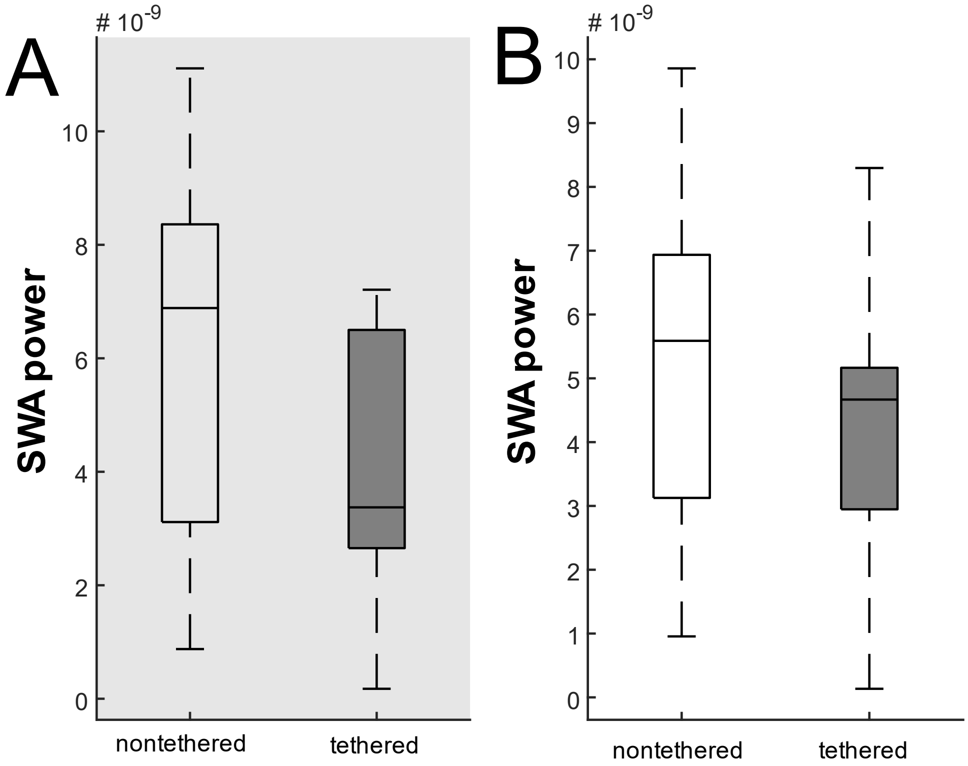

SWA power **on the first day** of experimental observation period for each 10-second episode that was scored NREM. **A:** SWA power for the **dark phase**. There was no significant difference between the nontethered (n = 6) and tethered (n = 7) animals. **B:** SWA power for the **light phase**. There was no significant difference between the nontethered (n = 6) and tethered (n = 7) animals. Box-plots show minimum to maximum values with median. Differences between the groups were tested using the Mann-Whitney-U test.

Supplementary Figure S11: Linear regression model of behavioral and biochemical parameter

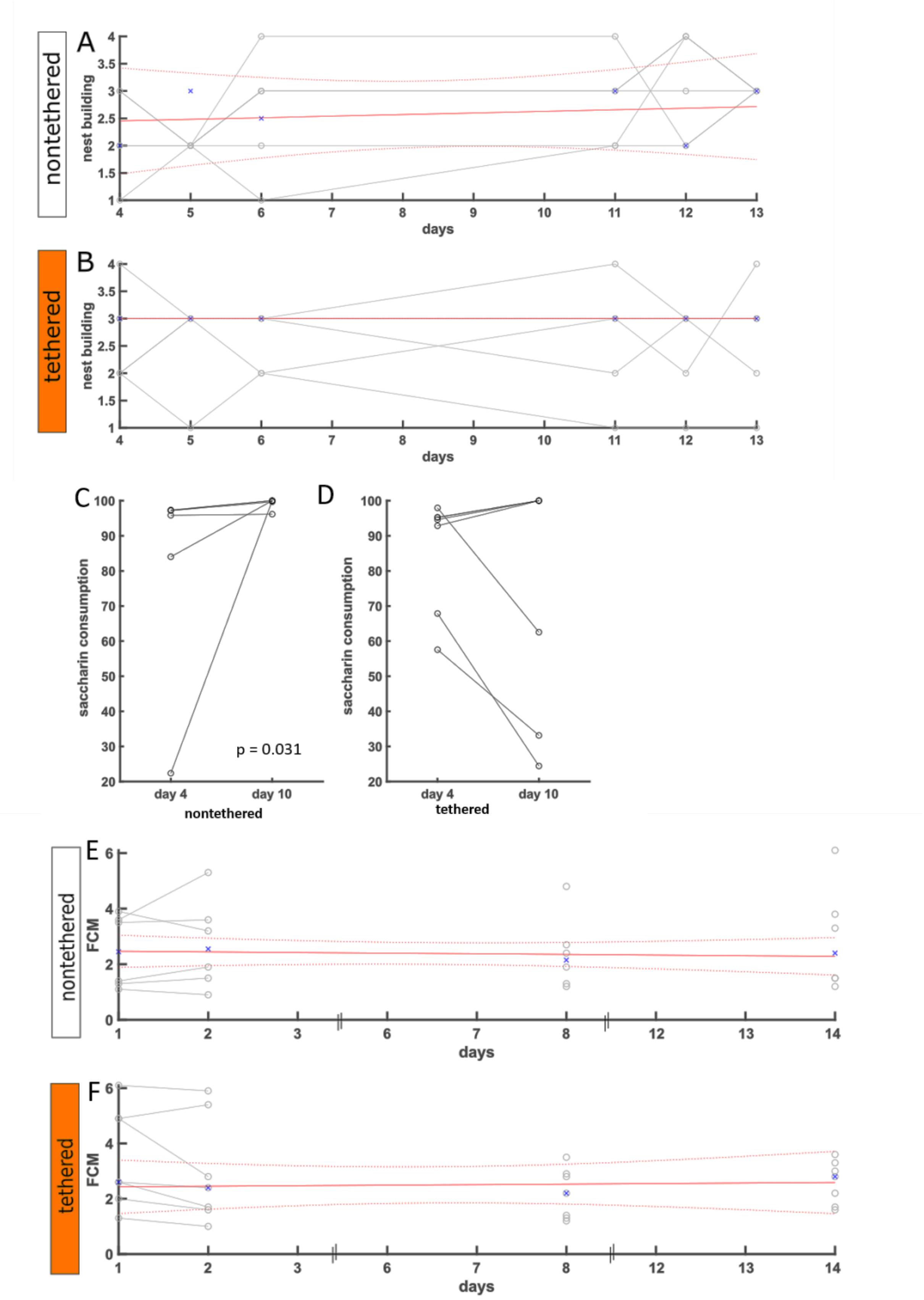

Linear regression model of behavioral and biochemical parameter of the nontethered (n = 6) and tethered (n = 7) animals throughout the experimental observation phase. For each animal, the average per day was calculated. The x-axis represents the days. **A, B**: Linear regression model of nest building for nontethered (A) and tethered (B) animals. No significant differences were detected over the time course of the observation period. **C, D**: Linear regression model for saccharin consumption for nontethered (C) and tethered (D) animals. The saccharin consumption in the nontethered animals increased significantly ($p = 0.031$) between week 1 and 2. **E, F**: Linear regression model for FCM for nontethered (E) and tethered (F) animals. No significant differences were detected over the time course of the observation period. Data are plotted individually for each animal.

Supplementary Figure S12: Averaged fecal corticosterone metabolite (FCMs)

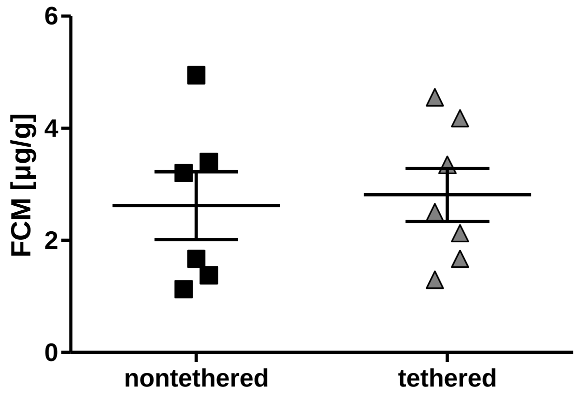

Averaged fecal corticosterone metabolite values throughout the experimental observation period. There was no significant difference between the nontethered (n = 6) and tethered (n = 7) animals. Data are presented as mean ± SEM.

Supplementary Table S1 and Supplementary Figure S13: Calculation of the AUC for sleep/wake transitions, SWA power, behavioral and biochemical parameter

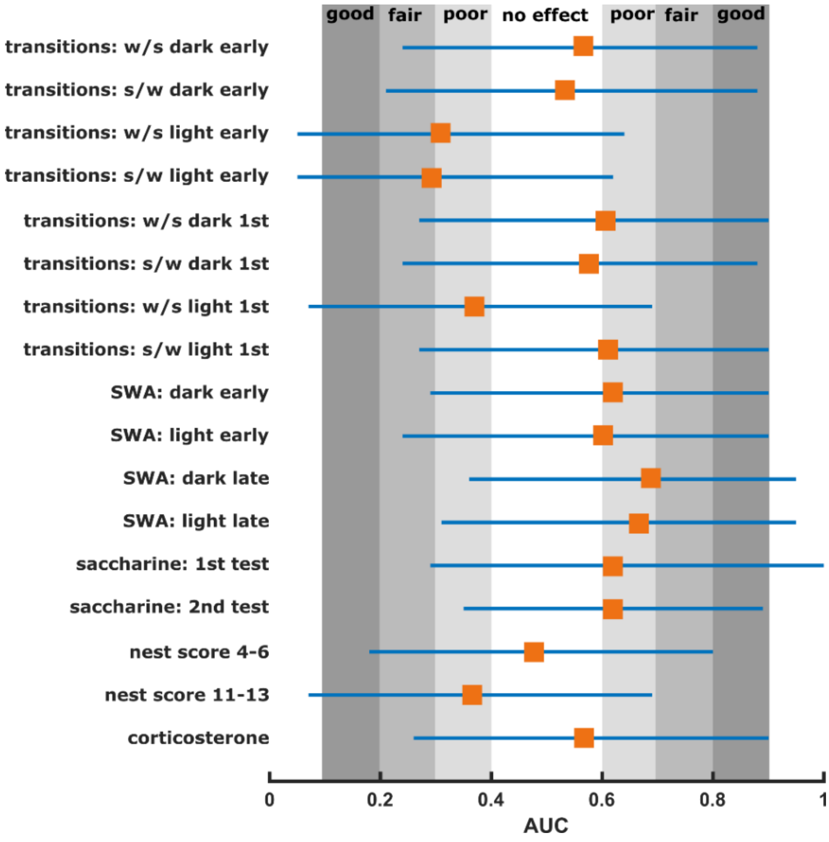

Figure		AUC	95% CI	
Figure5A	T: ws_dark_early	0.57	0.24	0.88
	T: sw_dark_early	0.54	0.21	0.88
Figure5B	T: ws_light_early	0.31	0.05	0.64
	T:sw_light_early	0.29	0.05	0.62
FigureS7	T: ws_dark_1st	0.61	0.27	0.9
	T: sw_dark_1st	0.58	0.24	0.88
	T: ws_light_1st	0.36	0.07	0.69
	T:sw_light_1st	0.61	0.27	0.9
Figure6A	SWA: dark_early	0.62	0.29	0.9
Figure6B	SWA: light_early	0.6	0.24	0.9
Figure6C	SWA: dark_late	0.69	0.36	0.95
Figure6D	SWA: light_late	0.67	0.31	0.95
Figure7A	nest1	0.48	0.18	0.8
Figure7B	nest2	0.36	0.07	0.69
Figure7C	saccharin 1	0.62	0.29	1
Figure7D	saccharin 2	0.62	0.35	0.89
Figure7F	serum corticosterone	0.58	0.26	0.9

Supplementary Figure S14: Activity analysis

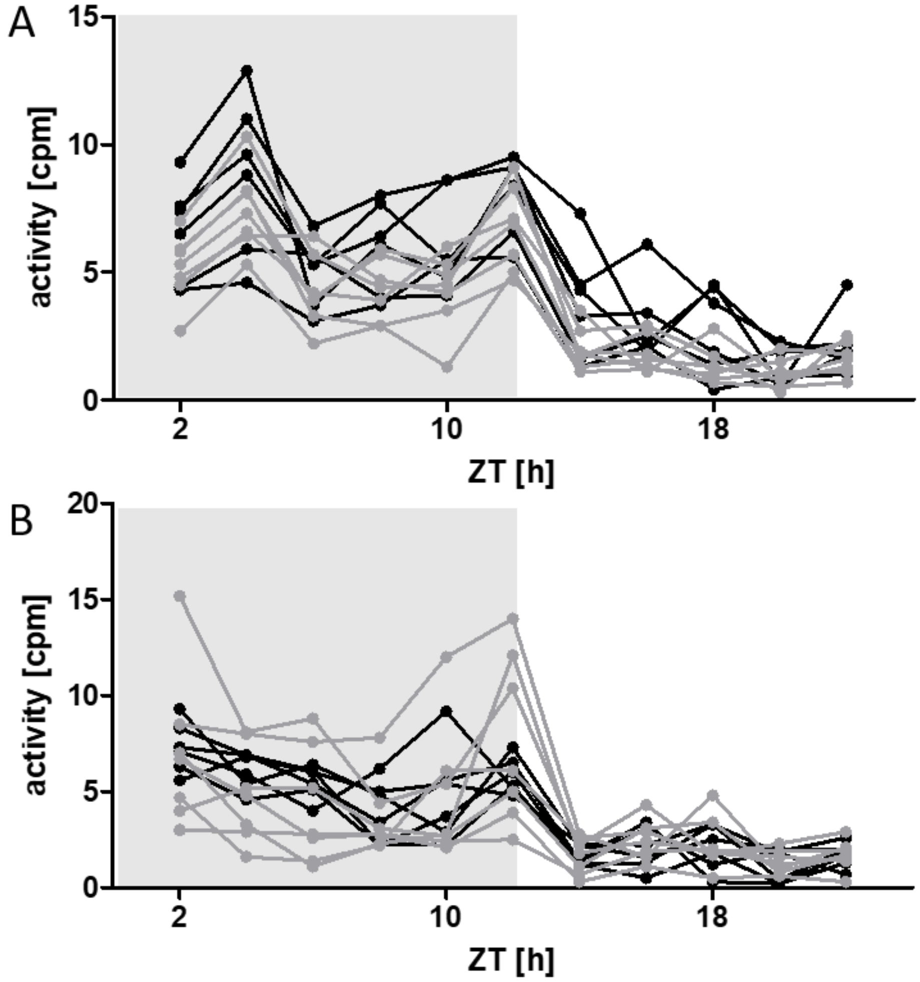

Activity analysis of the nontethered (n = 6) and tethered (n = 7) animals for nonoverlapping 2 h observation episodes throughout the 22 h. Lights were turned on after 12 hours. Gray exhibits the dark phase, white exhibits the light phase. The x-axis represents Zeitgeber time (ZT). cpm = counts per minute. **A.** Averaged **baseline** values for the nontethered (black) and tethered (gray) animals. The analysis detected no significant difference between the groups at any time point. **B**: Averaged values for the **first day** of the experimental observation period. The analysis detected no significant difference between the nontethered (black) and tethered (gray) animals at any time point. Data are plotted individually for each animal. Differences between the groups were tested using a two-way ANOVA with factors "nontethered/tethered" and "hours", followed by a post-hoc Bonferroni multiple comparison test.

References Supporting information

1 Jyoti, A., Plano, A., Riedel, G. & Platt, B. EEG, Activity, and Sleep Architecture in a Transgenic AβPP swe/PSEN1 A246E Alzheimer's Disease Mouse. *Journal of Alzheimer's Disease* **22**, 873-887 (2010).

2 Paxinos, G. & Watson, C. The rat brain in stereotaxic coordinates (New York: Academic Press, 2009).
